# Thermodynamic Temperatures of the Triple Points of Mercury and Gallium and in the Interval 217 *K* to 303 *K*

**DOI:** 10.6028/jres.104.002

**Published:** 1999-02-01

**Authors:** M. R. Moldover, S. J. Boyes, C. W. Meyer, A. R. H. Goodwin

**Affiliations:** National Institute of Standards and Technology, Gaithersburg, MD 20899-0001

**Keywords:** acoustic resonator, acoustic thermometry, argon, fixed point, gallium point, gas constant, ideal gas, mercury point, microwave resonator, resonator, speed of sound, spherical resonator, thermodynamic temperature, thermometry

## Abstract

We measured the acoustic resonance frequencies of an argon-filled spherical cavity and the microwave resonance frequencies of the same cavity when evacuated. The microwave data were used to deduce the thermal expansion of the cavity and the acoustic data were fitted to a temperature-pressure surface to deduce zero-pressure speed-of-sound ratios. The ratios determine (*T*–*T*_90_), the difference between the Kelvin thermodynamic temperature *T* and the temperature on the International Temperature Scale of 1990 (ITS-90). The acoustic data fall on six isotherms: 217.0950 K, 234.3156 K, 253.1500 K, 273.1600 K, 293.1300 K, and 302.9166 K and the standard uncertainties of (*T*−*T*_90_) average 0.6 mK, depending mostly upon the model fitted to the acoustic data. Without reference to ITS-90, the data redetermine the triple point of gallium *T*_g_ and the mercury point *T*_m_ with the results: *T*_g_/*T*_w_ = (1.108 951 6 ± 0.000 002 6) and *T*_m_/*T*_w_= (0.857 785 5 ± 0.000 002 0), where *T*_w_ = 273.16 K exactly. (All uncertainties are expressed as standard uncertainties.) The resonator was the same one that had been used to redetermine both the universal gas constant *R*, and *T*_g_. However, the present value of *T*_g_ is (4.3 ± 0.8) mK larger than that reported earlier. We suggest that the earlier redetermination of *T*_g_ was erroneous because a virtual leak within the resonator contaminated the argon used at *T*_g_ in that work. This suggestion is supported by new acoustic data taken when the resonator was filled with xenon. Fortunately, the virtual leak did not affect the redetermination of *R*. The present work results in many suggestions for improving primary acoustic thermometry to achieve sub-millikelvin uncertainties over a wide temperature range.

## 1. Introduction

In the Introduction, we briefly review the historical context of the present work, the conceptual basis of primary acoustic gas thermometry with spherical resonators, the present results, and the significant components of the uncertainty of these results.

### 1.1 Historical Context

Here we report new values for the thermodynamic temperatures of the triple points of mercury *T*_m_ and gallium *T*_g_ and the difference (*T*−*T*_90_) between the Kelvin thermodynamic temperature scale *T* and the International Temperature Scale of 1990 (ITS-90) from 217 K to 303 K. This work documents significant progress in a program at NIST/NBS to exploit spherical cavities for primary acoustic gas thermometry. This program began during 1978 when Moldover et al. [1] measured the frequencies of the acoustic resonances in an argon-filled spherical cavity and also deduced the radius of the cavity from the frequencies of microwave resonances within it. In doing so, they demonstrated the essential elements of primary acoustic thermometry using a spherical cavity. Important advances were made by Mehl and Moldover [2] and by Moldover, Mehl, and Greenspan [3] who published a detailed theory for the acoustic resonances of a nearly-spherical, gas-filled cavity as well as extensive experimental tests of the theory. These results guided Moldover et al. [4] in assembling a 3L, steel-walled, spherical cavity sealed with wax (the “gas-constant resonator”) which they used during 1986 to redetermine the universal gas constant *R* with a relative standard uncertainty of 1.7 × 10^−26^, a factor of 5 smaller than the uncertainty of the best previous measurement. Mehl and Moldover [5] also developed the theory of nearly-degenerate microwave resonances in a nearly-spherical cavity and showed how to use a few microwave resonances to deduce the volume of the cavity. Their theory was tested by Ewing et. al [6] who showed that a microwave measurement of the thermal expansion of the gas-constant resonator from 273 K to 303 K was consistent with a measurement based on mercury dilatometry.

The gas-constant resonator had not been optimized for the determination of the thermodynamic temperature *T*; however, it was very well characterized and it was available for measurements prior to the scheduled replacement of the International Practical Temperature Scale of 1968 (IPTS-68) with ITS-90. Thus, Moldover and Trusler [7] used the gas-constant resonator during 1986 to determine *T*_g_, the thermodynamic temperature of the triple point of gallium. Subsequently, during 1989, two of the present authors (M.R.M. and C.W.M.) used the gas-constant resonator to study the temperature scale in the range 213 K to 303 K and found evidence that the redetermination of *T*_g_ reported by Moldover and Trusler [7] was in error by approximately 4 mK [8].

**Fig. 1:**
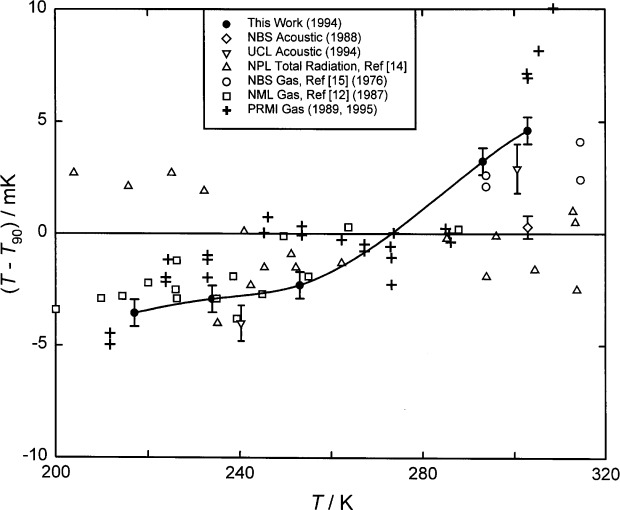
Comparison of the Kelvin thermodynamic temperature scale and ITS-90. The present data are designated “This Work (1994)” and the solid curve is a spline fit to them. The other data sources are: NBS gas thermometry, Ref. [15]; NML gas thermometry Ref. [12]; corrected PRMI gas thermometry, Ref. [13]; NPL total radiation thermometry, Ref. [14]; NBS acoustic thermometry, Ref. [7]; UCL acoustic thermometry, Ref. [11].

Here, we report the results of more recent measurements conducted with the gas-constant resonator, primarily during 1992, which lead to new values of (*T*−*T*_90_) on the five isotherms: 217.0950 K, 234.3156 K, 253.1500 K, 293.1300 K, 302.9166 K. One of these isotherms (302.9166 K) is very near *T*_g_ and another (234.3156 K) is very near *T*_m_, the thermodynamic temperature of the triple point of mercury. For the present data, the standard uncertainty of (*T*−*T*_90_) is approximately 0.6 mK, depending mostly upon the model fitted to the acoustic data. This sub-millikelvin uncertainty is much smaller than the uncertainties characteristic of other methods of primary thermometry in this temperature range. (See Fig. 1.)

The present value of *T*_g_ is (4.3 ± 0.8) mK larger than the Moldover-Trusler [7] value. (All uncertainties in this manuscript are expressed as standard uncertainties.) With the benefit of both additional data and hindsight, we *conjecture* that the Moldover-Trusler determination of *T*_g_ was erroneous because the argon used in their work was progressively contaminated, perhaps by virtual leak into the resonator. (Any volume that was sealed from the laboratory and was connected to the resonator by a path of low pumping speed would act as a virtual leak. If such a volume were exposed to a contaminating gas (e.g., air at 100 kPa), it would fill rapidly via Poiseuille flow. Subsequently, the volume would be a persistent source of contamination because it would take a very long time to empty via molecular flow at low pressure.) Below, we present some data acquired when the gas-constant resonator was filled with xenon that strongly support this conjecture. The need to *conjecture* calls attention to a significant weakness of the gas-constant resonator and the apparatus associated with it: there were no satisfactory provisions for detecting contamination of the thermometric gas after it had been admitted into the resonator. Fortunately, all of the results from the gas-constant resonator on the 273.16 K isotherm are mutually consistent; thus, there is no evidence that contamination was a problem during the re-determination of *R*.

At the conclusion of the present work, the gas-constant resonator was disassembled. The dimensions of its component hemispheres were re-measured. Now the hemispheres and the associated apparatus are being reconstructed to correct deficiencies uncovered in the present work and to extend the range of primary acoustic gas thermometry up to 750 K. Improvements to the apparatus and procedures include: (1) flowing the thermometric gas through the apparatus to reduce the residence time of the gas by a factor of 100, thereby reducing exposure to possible contamination; (2) tuning the ports that admit gas to the resonator [9] thereby attaining simultaneously a high acoustic impedance near radial resonances and a low flow impedance; (3) bakeable gas-handling system and transducers (no polymer seals) minimizing possible contamination; (4) analysis of the gas exiting the resonator via gas chromatography; (5) simultaneous measurements of acoustic and microwave resonance frequencies; (6) positioning the microwave coupling probes to optimize the resolution of the nearly-degenerate microwave resonance frequencies; (7) provisions for measuring possible horizontal temperature gradients in the resonator; (8) provisions for measurement of the resonator’s temperature on ITS-90 with up to five long-stemmed standard platinum resistance thermometers that can be conveniently inserted and removed from contact with the resonator; and (9) extended data runs to acquire data on many, relatively closely-spaced isotherms. (The gas constant apparatus had to be disassembled to calibrate the thermometers. Thus, extended data runs were precluded by the concern that the thermometers might drift between calibrations.) This list of projected improvements makes it clear that the present results will not comprise NIST’s final contribution to acoustic gas thermometry in the temperature range 217 K to 303 K or above.

### 1.2 Conceptual Basis of the Present Measurements

#### 1.2.1 Acoustic Gas Thermometry

Primary acoustic thermometry relies on the connection between the speed of sound in a gas and the thermodynamic temperature. Elementary considerations of hydrodynamics and the kinetic theory of dilute gases leads to relationships between the thermodynamic temperature *T*, the average kinetic energy *E* in one degree of freedom, and the speed of sound *u*:

$$3E=\frac{1}{2}mv_{\mathrm{rms}}^{2}=\frac{3}{2}kT,\;u^{2}=\frac{\gamma }{3}v_{\mathrm{rms}}^{2}.$$ (1)

Here, *v*_rms_ is the root mean square speed of a gas molecule, *m* is its mass, *k* is the Boltzmann constant, and *g* is the ratio of the constant pressure to constant volume specific heat capacities which is exactly 5/3 for perfect monatomic gases. The International System of Units assigns the exact value 273.16 K to the temperature of the triple point of water *T*_w_. From this assignment and from Eqs. (1), the Kelvin thermodynamic temperature *T* of a gas can be determined from the zero-pressure limit of the ratio of speed of sound measurements at *T* and *T*_w_ with the equation

$$\frac{T}{273.16\;K}=\begin{array}{c} \lim\\ p\to 0 \end{array}\left(\frac{u^{2}\left(p,T\right)}{u^{2}\left(p,T_{w}\right)}\right).$$ (2)

In principle, Eq. (2) could be used to calibrate thermometers on the Kelvin thermodynamic scale and ultimately, this may become accepted practice.

Today, some useful implementations of Eq. (2) can be accomplished with stable thermometers that have never been calibrated using ITS-90. For example, in this work we could have used uncalibrated (“transfer-standard”) thermometers to redetermine the thermodynamic temperature of the triple point of mercury *T*_m_ (and of other fixed points). To do so, we would have recorded the indications of the uncalibrated thermometer when it was in a mercury-point cell and when it was at *T*_w_. Subsequently, the thermometer’s indications would be used to help adjust the temperature of the thermometric gas to *T*_m_ and then to *T*_w_ where *u*^2^(*p*,*T*_m_) and *u*^2^(*p*,*T*_w_) would be measured. Finally Eq. (2) would be applied. In practice we used standard platinum resistance thermometers (SPRTs) that had been calibrated on ITS-90 to adjust the temperature of the argon. We used SPRTs for two reasons. First, SPRTs are known to be very stable and second, our results at temperatures between fixed points could be preserved and reported as the difference (*T*−*T*_90_) as a function of *T* or of *T*_90_.

#### 1.2.2 Acoustic and Microwave Resonances in a Spherical Cavity

In this work, the speeds of sound in argon and in xenon were deduced from measurements of ***F***_a_, the frequencies of the radially-symmetric acoustic modes in the gas-constant resonator. (Conventionally, ***F***_a_ = *f*_a_ + *ig*_a_ is a complex number such that *f*_a_ is the center frequency of the resonance and *g*_a_ is the half-width of the resonance. In the context of frequency measurements, the subscripts “a” and “m” are used to distinguish acoustic modes from microwave modes; in the context of triple-point temperatures, the subscripts “a” and “m” identify argon and mercury, respectively.) There is a well-developed theory for the radially-symmetric acoustic modes that has been confirmed by detailed experiments [2], [4], [10]. The frequencies of these modes are only weakly sensitive to deformations of the shape of the cavity from a perfect sphere so long as the volume of cavity *V*(*p*,*T*) is unchanged. Thus, accurate measurements of *u*^2^(*p*,*T*) do not require accurate measurements of the shape of the cavity. They do require an accurate measurement of *V*(*p*,*T*), which is usually much easier. (In Ref. [4], the very small pressure dependence of *V*(*p*,*T*) was calculated and checked by a measurement. For the remainder of this Introduction, the pressure dependence of *V* will be ignored.) We write *u*(*p*,*T*) in terms of the frequencies and the volume,

$$u(p,T)=[f_{a}(p,T)+\Delta f_{a}(p,T)]\times {\left[V(T)\right]}^{1/3}/\Lambda_{a}.$$ (3)

In Eq. (3), the term Δ*f*_a_(*p*,*T*) represents several small, theoretically based, mode dependent corrections which must be added to the measured resonance frequencies and which depend mostly upon the boundary conditions. The constant ***Λ***_a_ is known exactly; it is an acoustic eigenvalue that depends upon the mode for which *f*_a_(*p*,*T*) is measured.

As shown in Ref. [5], the microwave modes within a nearly-spherical cavity occur in nearly-degenerate multiplets with 2*l* + 1 components. (*l* is a positive integer.) The frequency of each component of a multiplet depends upon the details of the shape of the cavity; however, the average frequency of each multiplet is not sensitive to smooth deformations of the cavity that leave its volume unchanged. In analogy with Eq. (3), the speed of light in the gas *c*(*p*,*T*) can be related to the frequencies of the microwave resonances ***F*_m_** = *f*_m_ + *ig*_m_ through

$$c(p,T)=\langle f_{m}(p,T)+\Delta f_{m}(p,T)\rangle \times {\left[V(T)\right]}^{1/3}/\Lambda_{m}.$$ (4)

In Eq. (4), the term Δ*f*_m_(*p*,*T*) represents the mode-dependent corrections that must be added to the measured resonance frequencies, *Λ*_m_ is an eigenvalue for the microwave multiplet chosen, and the brackets “ 〈…〉” denote the average over the components in a multiplet.

One can combine Eq. (3) and Eq. (4) to eliminate *V*(*T*) and obtain

$$\frac{u(p,T)}{c(p,T)}=\frac{f_{a}(p,T)+\Delta f_{a}(p,T)}{\langle f_{m}(p,T)+\Delta f_{m}(p,T)\rangle }\times \frac{\Lambda_{m}}{\Lambda_{a}}.$$ (5)

For primary thermometry in an ideal situation, Eq. (5) is used at the temperature *T* and at *T*_w_ and the zero-pressure limit is taken at each temperature. This leads to an idealized working equation:

$$\frac{T}{273.16\;K}=\begin{array}{c} \lim\\ p\to 0 \end{array}{\left(\frac{f_{a}(p,T)+\Delta f_{a}(p,T)}{\langle f_{m}(p,T)+\Delta f_{m}(p,T)\rangle }\right)}^{2}\times \begin{array}{c} \lim\\ p\to 0 \end{array}{\left(\frac{\langle f_{m}(p,T_{w})+\Delta f_{m}(p,T_{w})\rangle }{f_{a}(p,T_{w})+\Delta f_{a}(p,T_{w})}\right)}^{2}.$$ (6)

As implied by the absence of *V*(*T*) in Eqs. (5) and (6), and as emphasized by Mehl and Moldover [5], the spherical cavity plays a limited role in measuring *u*/*c*, the ratio of the speed of sound to the speed of light. One may view the cavity as a temporary artifact that must remain dimensionally stable just long enough to measure *f*_a_(*p*) and $\langle f_{m}(p)\rangle$ at the temperature *T* and that must not change its shape (and eigenvalues) too much when the frequency measurements are repeated at *T*_w_. (Small, smooth changes in the shape of the cavity affect the eigenvalues only in the second order of the small change.) Although Eq. (6) fully accounts for the change of the cavity’s volume upon going from *T* to *T*_w_, the computation of the correction terms Δ*f*_a_(*p*,*T*) and Δ*f*_m_(*p*,*T*) require moderately accurate knowledge of the cavity’s dimensions, the electrical conductivity and the mechanical compliance of the shell bounding the cavity, and other properties of the both resonator and the thermometric gas.

#### 1.2.3 Particulars of This Work

Unfortunately, the gas-constant resonator did not have provision for simultaneous acoustic and microwave measurements that would be required to use Eq. (6). Therefore, as in Ref. [6], *V*(*T*) was deduced from measurements of *f*_m_(*T*) made when the cavity was evacuated and *V*(*T*) was assumed to be a reproducible function of *T* for intervals of weeks. This assumption is supported below by the important observation that the values of *f*_a_(*p*,*T*_w_) that were obtained during the measurement of *R* in 1986, the measurement of *T*_g_ in 1987, and subsequent thermometry in 1989 and in 1992 (this work) agree within 1 part in 10^6^.

In principle, *u* (and *T*) could be determined from measurements of the frequencies of a single acoustic mode and a single microwave triplet. In this work, as in the redetermination of *R*, five non-degenerate acoustic modes spanning a frequency ratio of 3.8 : 1 were used. Also, three microwave triplets spanning a frequency ratio of 3.4 : 1 were used. The redundant acoustic and microwave measurements were used to determine some components of the uncertainty in measuring (*T*−*T*_90_) and to search for limitations of the theories for the corrections Δ*f*_a_ and Δ*f*_m_.

The microwave measurements avoid an assumption that is often made in gas thermometry; specifically, that the volumetric expansion of an assembled cavity is identical with that computed from the measured linear thermal expansion of other artifacts made from the same material. In fact, the present microwave measurements provide evidence that the expansion of the gas-constant resonator was anisotropic, an effect that would not have been detected by measurements of the thermal expansion of one dimension of an artifact made of the same metal.

The extrapolation of *f*_a_ to zero pressure was accomplished by fitting the acoustic data on all the isotherms simultaneously to polynomial functions of the pressure with temperature-dependent coefficients. This procedure together with the practice of measuring *f*_m_ when the resonator was evacuated and the assumption that the dimensions of the gas-constant resonator were stable in time leads to a modification of Eq. (6) that more nearly represents the working equation used in this work:

$$\frac{T}{273.16\;K}=\begin{array}{c} \lim\\ p\to 0 \end{array}{\left(\frac{f_{a}(p,T)+\Delta f_{a}(p,T)}{f_{a}(p,T_{w})+\Delta f_{a}(p,T_{w})}\right)}^{2}\times {\left(\frac{\langle f_{m}(T_{w})+\Delta f_{m}(T_{w})\rangle }{\langle f_{m}(T)+\Delta f_{m}(T)\rangle }\right)}^{2}.$$ (7)

Typically, a single measurement of *f*_a_ with the gas-constant resonator had a repeatability of 0.2 × 10^−6^ *f*_a_ corresponding to 0.1 mK at 300 K. The repeatability measurement of *f*_m_ was less than 0.1 × 10^−6^ *f*_m_. The excellent repeatability of these frequency measurements indicates that acoustic gas thermometry has the potential to be very accurate. The larger uncertainties encountered in this work are listed in Sec. 1.4 and their evaluation is discussed throughout the body of this manuscript.

### 1.3 Results

#### 1.3.1 The Differences (*T*−*T*_90_)

The present results for (*T*−*T*_90_) are listed in Table 1 and they are compared with other results in the same temperature range in Fig. 1. Included in Fig. 1 are results from primary acoustic thermometry at University College London (labeled “UCL Acoustic”) at 240 K and 300 K. The UCL results agree with the present results within the remarkably small combined uncertainties. At the time of this writing, the UCL results have not been published [11]. They are a product of research that started after the NIST/NBS program had begun and the techniques used at UCL are described in connection with a calculation by Ewing et al. [10] of the effects on acoustic resonance frequencies of imperfect thermal accommodation at the shell-gas boundary.

Fig. 1 includes data that were used to establish ITS-90. Among these, the present results below the triple point of water (*T*_w_) agree best with the NML gas thermometry [12], and with the corrected PRMI gas thermometry [13]. (Before the 1995 correction, the PRMI gas thermometry results were much closer to the *T*_90_ baseline.) The data on Fig. 1 labeled “NPL Total Radiation” came from Ref. [14]; the data labeled “NBS Gas” came from Ref. [15].

In Fig. 1, the Moldover-Trusler [7] redetermination of *T*_g_ is labeled “NBS Acoustic, 1988.” The difference between this value and the present result is (4.3 ± 0.8) mK. As mentioned above, we conjecture that the Moldover-Trusler result was erroneous because a virtual leak contaminated the argon used in their work. In Sec. 8.4 below, we discuss the evidence supporting this conjecture and we present some data acquired when the gas-constant resonator was filled with xenon that also support this conjecture.

Table 1 summarizes the present results for (*T*−*T*_90_) that were obtained using argon and xenon. The columns labeled “isotherm fits” and “surface fit” resulted from two different analyses of the acoustic data for argon. For the isotherm fits, the squares of speeds of sound *u*^2^ on each of the five isotherms listed as well as the isotherm 273.16 K were fitted by polynomial functions of the pressure *p*. In this analysis, 24 parameters were used to represent the six isotherms comprising the *u*^2^(*p*,*T*) surface. For the surface fit, three constraints were imposed on the *u*^2^(*p*,*T*) surface, leaving only 11 or 12 parameters to be fit by the same data. The constraints applying to the temperature range 217 K ≤ *T* ≤ 303 K were: (1) the difference Δ*A*_1_(*T*) ≡ *A*_1_(*T*,expt.) − *A*_1_(*T*,calc.) must be an empirical linear function [here, *A*_1_(*T*,expt.) is the coefficient of *p* in the polynomial and *A*_1_(*T*,calc.) is the prediction from a semi-empirical interatomic potential taken from the literature], (2) the coefficient *A*_2_(*T*) of *p*^2^ in the polynomial is represented as an empirical quadratic function of 1/*T*, and (3) the thermal accommodation coefficient *h* is exactly one. Constraints (1) and (2) are plausible because the present isotherms are well above the critical temperature of argon (1.4 ≤ *T*/*T*_c_ ≤ 2.0) and well below the critical density (*ρ*/*ρ*_c_ ≤ 0.02) where the virial coefficients of argon are only weakly temperature dependent. The third constraint is the temperature-independent upper bound to *h*, which will be discussed in Sec. 8.3.

As expected, the constrained (surface) fits resulted in smaller statistical uncertainties of the parameters; however, Table 1 shows that the constrained and unconstrained fits yield consistent results for (*T*−*T*_90_). Furthermore, the standard deviations of the fits *σ*(*u*^2^) relative to 〈*u*_0_^2^〉 were comparable. (For the constrained fit, 10^6^ *σ*(*u*^2^)/〈*u*_0_^2^〉 = 1.12; for the isotherm fits, 0.83 ≤ 10^6^ *σ*(*u*^2^)/〈*u*_0_^2^〉 ≤ 1.50.) Thus, the experimental evidence does not contradict the constraints. Remarkably, the constrained fit leads to values of (*T*−*T*_90_) that average 0.6 mK larger than the unconstrained fit. Imposing the constraints increases the zero-pressure limit of *u*^2^(*p*,*T*) on all of the isotherms except the 273.16 K isotherm. We cannot explain this; however, in our opinion, it is advantageous that the constrained fit does not give the speed-of-sound data on the 273.16 K isotherm a privileged role such that they affect all of the values of (*T*−*T*_90_).

We considered a variety of additional fits (Sec. 8.4) including a constrained fit in which Δ*A*_1_(*T*) was represented as a quadratic function of *T*, and both constrained and unconstrained fits excluding acoustic data for the (0,6) mode, the mode that deviated most from the average of the other modes. These analyses led to values of (*T*−*T*_90_) that were either between the constrained and unconstrained results or very close to them. Obviously, the acoustic data could have been analyzed with many other combinations of constraints. The data are tabulated in appendices, allowing the reader to impose the constraints that he/she prefers.

In view of these factors, we recommend the values of (*T*−*T*_90_) resulting from the constrained fit; however, the values are model-dependent. With some arbitrariness, we recommend enlarging the standard uncertainty of (*T*−*T*_90_) to 0.6 mK. The value 0.6 mK encompasses the extreme values of (*T*−*T*_90_) resulting from the models that we investigated and is approximately twice the uncertainties resulting from the surface fits.

We conclude this discussion of the results by noting that the recommended uncertainty of (*T*−*T*_90_), namely, 0.6 mK, is very small in the context of results obtained by other methods, as shown in Fig. 1.

#### 1.3.2 Triple Points of Gallium and Mercury

The isotherms near the triple points of gallium *T*_g_, mercury *T*_m_, and water *T*_w_ have a special status. For these isotherms, our platinum thermometers were used to indicate when the temperature of the resonator had been adjusted to be equal to the temperature of one of these triple points. For this very limited function, the thermometers had to be stable; however, they did not ever have to be calibrated on ITS-90. Thus, the present acoustic and microwave data redetermine *T*_g_ and *T*_m_ without reference to ITS-90. Using Eq. (7) with the microwave data and the constrained fits to the acoustic data leads to the results *T*_g_/*T*_w_ = (1.108 951 6 ± 0.000 002 6) and *T*_m_/*T*_w_ = (0.857 785 5 ± 0.000 002 0) which determine both *T*_g_ and *T*_m_ through the definition: *T*_w_ ≡ 273.16 K, exactly.

### 1.4 Components of the Uncertainties

Table 2 lists the important components of the standard uncertainty (*u*_s_) in the determination of (*T*−*T*_90_)/*T* from the measurements of the quantities in Eq. (7). The evaluation of these contributions is a major portion of the body of this manuscript. Here, we outline the phenomena that contributed to *u*_s_.

#### 1.4.1 Microwave Measurements

To determine *T*, we required the combination of microwave frequencies [〈*f*_m_(*T*_w_)+Δ*f*_m_(*T*_w_)〉/〈*f*_m_(*T*)+Δ*f*_m_(*T*)〉]^2^ which equals the ratio [*a*(*T*)/*a*(*T*_w_)]^2^ when *a*(*T*) is defined to be the average radius of the spherical cavity such that *V*_0_(*T*) ≡ (4/3)π[*a*(*T*)]^3^ and where *V*_0_(*T*) is the zero-pressure limit of the volume. The ratio [*a*(*T*)/*a*(*T*_w_)]^2^ was computed from a polynomial function of the temperature that had been fitted to the data for 〈*f*_m_(*T*) + Δ*f*_m_(*T*)〉. We denote the relative standard uncertainty of *a*(*T*) by *u*_r_(*a*). The primary components of *u*_r_(*a*) arise from the different values of 〈*f*_m_(*T*) + Δ*f*_m_(*T*)〉 for the three microwave triplets studied and from the uncertainties in the correction term Δ*f*_m_(*T*). The differences among the triplets account for Row 1 of Table 2. Theoretically, the correction term Δ*f*_m_(*T*) is proportional to *δ*_m_(*T*), the microwave “penetration depth” or “skin depth.” Two methods were used to obtain *δ*_m_ (*T*). For the first method, *δ*_m_ (*T*) was computed from published values for the electrical resistivity of stainless steel and Row 2 of Table 2 accounts for the uncertainties in adapting the dc resistivity data to the present circumstances (Sec. 3.2.2.). This approach sets a lower bound to *δ*_m_(*T*). A plausible upper bound for *δ*_m_ (*T*) was calculated from the measured half-widths *g*_m_ of the microwave multiplets. (The lower-bound values of *δ*_m_(*T*) were used in computing Δ*f*_m_(*T*) for determining the temperature in Table 1; if the upper-bound had been used, *T*/*T*_w_ would have been changed by less than 0.3 × 10^−6^.) The differences between the upper and lower bounds of *δ*_m_(*T*) were used to calculate a contribution to the standard uncertainty *u*_s_(*T*) which, expressed as a fraction of (*T*−*T*_90_)/*T*, appears in Row 3 of Table 2. Negligible (< 0.1 × 10^−6^) contributions to *u*_r_(*a*) came from the uncertainty of each measurement of *f*_m_ and from the uncertainty of the temperature during the measurement of *f*_m_(*T*).

#### 1.4.2 Acoustic Measurements

The relative uncertainty in the measurement of an acoustic resonance frequency was usually less than 0.3 × 10^−6^. With limited and well understood exceptions, the five acoustic modes yield results that are consistent at this level. However, the zero pressure limit of the speed of sound was determined by fitting a function of pressure to the measured resonance frequencies. The correlations among these parameters contribute to the uncertainty in the speed of sound ratios accounting for the different uncertainties resulting from the isotherm fits (Table 2, Rows 4 and 5) and the surface fit (Table 2, Rows 6 and 7).

#### 1.4.3 Temperature Measurements

In the present work, three capsule-style standard platinum resistance thermometers were calibrated at the triple points of argon, mercury, water, and gallium (*T*_a_, *T*_m_, *T*_w_, *T*_g_) and then installed in the resonator. The uncertainty of the thermometry resulted from the uncertainty of each calibration measurement (Table 2, Row 8) and from drifts in the system (thermometers + resistance bridge + standard resistor) during the weeks between calibrations. The latter was estimated (Table 2, Row 9) from the change of the calibrations during the interval in which the acoustic measurements were made. Additional uncertainty resulted from the small temperature difference between the thermometers embedded in the top (“north pole”) and bottom (“south pole”) of the spherical shell. The difference never exceeded 0.5 mK. We estimate that our imperfect knowledge of the volume average of the temperature distribution within the thermometric gas is no more than 0.1 mK relative to the thermometer calibrations. This effect accounts for Row 10 of Table 2. Except at the calibration temperatures, the non-uniqueness of ITS-90 contributed to the uncertainty of the temperature measurements. (Table 2, Row 11)

The “additional sources” of uncertainty in Table 2 arise from the uncertainty of the thermal conductivity of the argon and from imperfect pressure measurements. They are considered elsewhere in this manuscript.

### 1.5 Organization of This Manuscript

The remainder of this manuscript is organized into major sections as follows: Sec. 2, modifications of the apparatus and the experimental procedures; Sec. 3, microwave measurements and their interpretation; Sec. 4, acoustic measurements; Sec. 5, thermometry; Sec. 6, characterization of the gases; Sec. 7, pressure and other thermophysical quantities; Sec. 8, analysis of the acoustic results; Sec. 9, comparisons of acoustic results with previous results; Sec. 10, other tests for systematic errors; Sec. 11, tabulated data; and Sec. 12, references.

## 2. Apparatus and Procedures

**Fig. 2:**
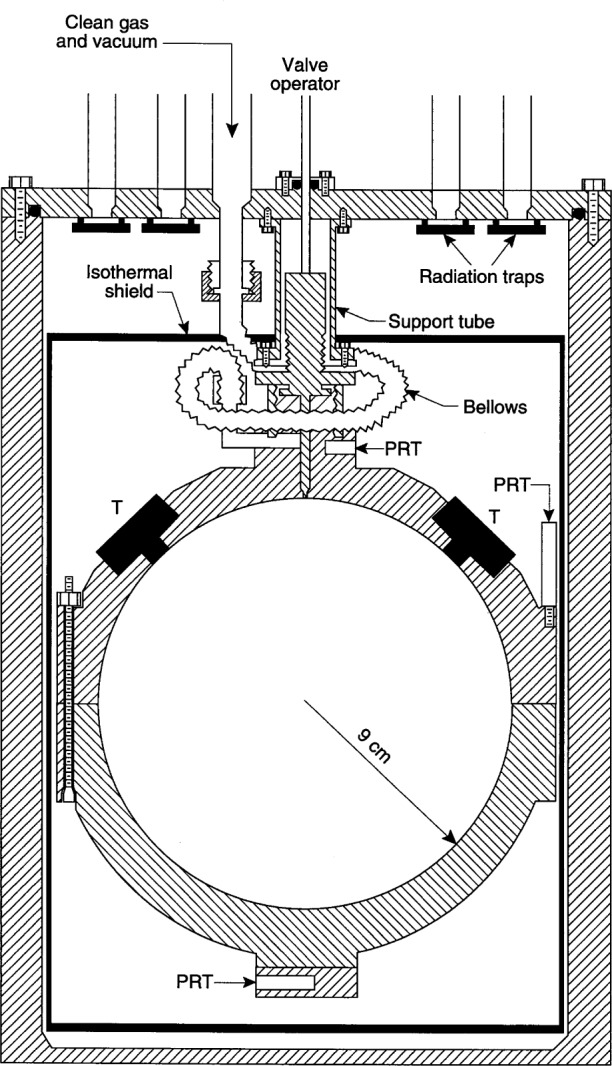
Schematic cross-section of the gas-constant resonator and pressure vessel.

The apparatus and procedures used have been described in extensive detail in earlier publications. [4,7] These details will not be repeated here; instead, we discuss the few modifications that were required to improve the thermostatting for the present work and the results of the dimensional measurements that were made when the apparatus was disassembled at the conclusion of the present work. Figure 2 shows a cross-section of the gas-constant resonator and the pressure vessel that enclosed it. The pressure vessel was immersed in an insulated, thermostatted, well-stirred, methanol bath.

### 2.1 Modifications to the Apparatus

**Fig. 3:**
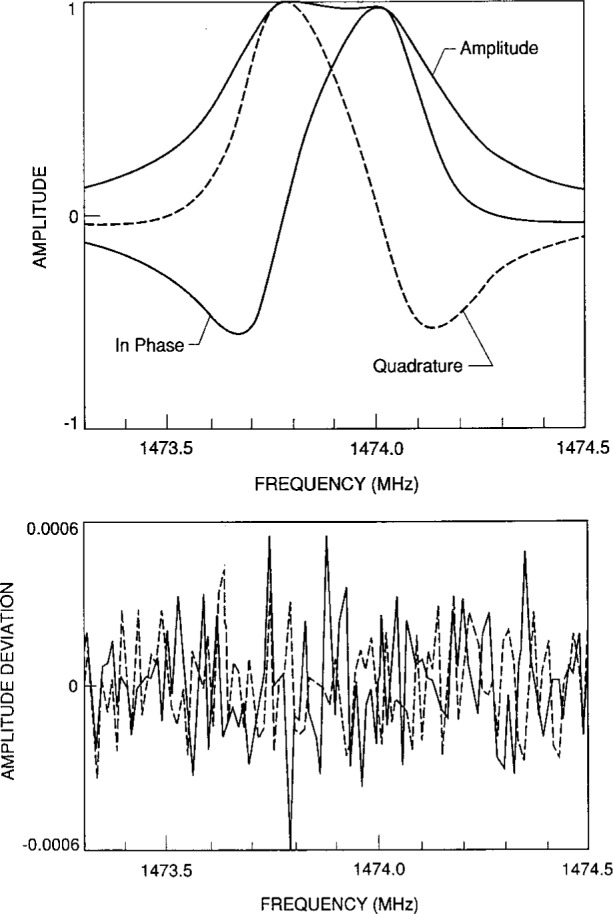
Top: Measurements of the in phase and quadrature signals detected as the microwave generator was swept through the TM11 “triplet.” Bottom: Deviations of the detected signals from a fit of Eq. (9) to the data in the upper panel. In this particular case, the fit determined 10 parameters: two complex resonance frequencies, two complex amplitudes, and a complex additive constant.

Several modifications were made to improve the thermostatting of the gas-constant resonator. When the gas constant was measured, a 2 cm long bellows led from the isolation valve atop the resonator to the gas handling system. (See Figs. 3 and 5 in Ref. [4].) Subsequently, during the manipulations associated with the microwave measurements, the bellows was damaged. Prior to the acoustic measurements of 1989 the bellows was replaced with a 23 cm long, copper tube that had been bent into a circle and placed in a horizontal plane nearly concentric with the valve atop the resonator.

During preliminary measurements performed in 1989, we found that as the temperature of the methanol bath was reduced below ambient temperature, the vertical temperature gradient across the resonator increased. When the methanol reached 213 K, the top of the resonator was 3 mK warmer than the bottom suggesting that a thermal link from the top to ambient temperature existed. This gradient was *not* reduced by three changes: (1) thinning the supports of the pressure vessel, (2) improving the radiation shields in the tubes leading to the resonator and, (3) improving the stirring of the bath.

However, the gradient was reduced to about 1 mK by surrounding the resonator with a cylindrical heat shield comprised of 3 mm thick copper strips. The strips were separated from each other but all were thermally anchored to the top and bottom of the resonator with thick aluminum strips. The shield was insulated from the walls of the pressure vessel by a 3 mm wide space filled with argon. This arrangement was used for the microwave measurements of the thermal expansion; however, the 1 mK gradient at 213 K was larger than desired for the acoustic measurements.

Immediately prior to the measurements of 1992 three additional changes were made to reduce the gradient: (1) The copper tube was replaced with a 23 cm long, thin-walled, stainless steel bellows (see Fig. 2) which was mounted in the same manner as had been the copper tube; (2) closed cell foam insulation was glued to the underside of the aluminum plate atop of the fluid bath to reduce the temperature gradient within the bath itself; and (3) a new shield, constructed entirely of copper, was installed. This shield, labeled “Isothermal shield” in Fig. 2, was comprised of a 2 mm thick cylinder and two 5 mm thick end plates. The shield was suspended from the central support tube, but otherwise was not in contact with either the resonator or the pressure vessel. All wires and electrical leads were thermally anchored to the top of the shield as well as the top-plate of the pressure-vessel. These modifications reduced the temperature difference between the top and the bottom of the resonator to less than 0.5 mK at all temperatures. Further discussion of temperature gradients appears in Sec. 5.3.

The lowest temperature at which the gas-constant apparatus could be used for primary thermometry was determined by the O-ring that was used to seal the body of the pressure vessel to its lid. When the temperature was too low, this seal leaked and either the methanol flowed from the bath into the pressure vessel or the gas from the pressure vessel flowed into the bath. The Viton^3^ O-rings that had been used were replaced with silicone rubber O-rings since they have a lower ultimate working temperature than their Viton equivalents. Even with this change, the O-ring leaked at the lowest temperature of study, restricting the maximum pressure to 360 kPa on the 217 K isotherm. The Viton O-rings that sealed the transducers in their ports within the resonator were not replaced. Usually, the pressure difference across these O-rings was only 1 kPa or less, with the higher pressure inside the resonator. If these O-rings were leaking significantly during the intervals that the valve atop the resonator was closed, the resonance frequencies would have shown a systematic time dependence that was not detected.

### 2.2 Dimensional Measurements

The fabrication and characterization of the gas-constant resonator were described in detail in [4]. As discussed in 1988 in Sec. 3.7 of Ref. [4], it was not possible at that time to obtain a satisfactory interpretation of the frequency-splittings of the non-radial (1,3) and (1,8) acoustic modes in terms of the geometry of the assembled resonator. (For the acoustic modes, the notation (*l*,*n*) is used to identify the order of the spherical Bessel function (*l* = 0, 1, 2, …) and *n* denotes the number of nodes in the radial component of the acoustic velocity.) The problem persisted after measurements of the frequencies of microwave resonances in the same cavity [6,7]. We now reconsider this problem.

The dimensions of each hemisphere have been measured three times. The first series of measurements was made by the NBS shop that fabricated the hemispheres using their coordinate measuring machine. These measurements were completed before the resonator was assembled in 1985 and the resulting data are denoted R1985 in Table 3. In Table 3, three dimensions are given for each hemisphere: (1) as in Fig. 2 of Ref. [4], *R*_c_ is the exterior radius of the cylindrical surface near the equator, (2) *R*_s_ is the average interior radius of the nearly spherical surface, and (3) *h*_s_ is the length of the cylindrical extension of each hemisphere beyond the plane that would terminate a perfect hemisphere.

After the resonator was disassembled in 1997, the second series of measurements, denoted P1997, were made by the Precision Measurement Division of NIST. These measurements used their coordinate measuring machine, which was the most accurate machine available to us. We were surprised by the comparatively large differences between the R1985 and P1997 results; therefore, we had a third series of measurements denoted F1997 made by the Fabrication Technology Division of NIST using a third coordinate measuring machine. (The machine used in 1985 was no longer available.)

The F1997 measurements are consistent with the nominally more accurate P1997 measurements. As indicated in Table 3, the average radius of each hemisphere *R*_s_ was approximately 0.02 mm smaller than reported in Ref. [4] and the average radius of the cylindrical boss *R*_c_ on each hemisphere was approximately 0.01 mm larger than reported in Ref. [4]. These differences are larger than the uncertainty of 0.005 mm claimed for the R1985 data and we have no explanation for the differences. Fortunately, these dimensions were not critical for either the present study of ITS-90 or for the redetermination of the universal gas constant *R*. The cylindrical bosses were used to align the hemispheres during the assembly of the resonator. The R1985 and P1997 values for *R*_c_ differ; however, both series of measurements show that *R*_c_ of both hemispheres was nearly identical. This was confirmed by simple observations made with the hemispheres in contact with each other.

The P1997 measurements did confirm that essentially all of the cylindrical extension of the hemispheres had been removed prior to the assembly of the resonator. This had been in doubt after the resonator was first assembled [4].

The disassembly of the resonator in 1997 revealed another surprise. When the transducers were inserted into their ports in the upper hemisphere of the resonator, the diaphragms of the microphones were not aligned with the interior surface of the hemisphere as intended. Instead, the transducers were recessed approximately 0.9 mm in their ports. Thus, when the resonator was assembled, the interior of the upper hemisphere had two coin-shaped volumes 9.49 mm in diameter and 0.9 mm deep. Measured by total volume, these coin-shaped cavities were smaller departures from the intended spherical figure than either the cylindrical extensions to the equators of the hemispheres or the difference between the radii of the hemispheres. The upper hemisphere and both transducer assemblies were machined in accordance with their drawings. We surmise that a design error was made, one surface of a 0.89 mm high locating step was used as the reference for designing the transducer ports and, by accident, the other surface of the step was used as the reference for designing the transducer assemblies. This error in design is similar to the error made during the ground tests of the mirror for the Hubble Space Telescope.

The unintentional and unknown presence of the coin-shaped volumes did not compromise the redetermination of *R*. For that work, the resonator’s volume was determined by weighing the mercury required to fill it. During the weighings, plugs were substituted for the transducers and the coin shaped volumes were present and accounted for. See Ref. [4] for details.

In summary, the cavity differed from a spherical figure in three respects: (1) the two hemispheres had different radii, (2) both hemispheres had cylindrical extensions, and (3) there were two coin-shaped recesses in the upper hemisphere. These departures from sphericity partially removed the degeneracy of the microwave modes [6] and of the non-radial acoustic modes [4].

Mehl [16],[17] expanded the shape of a cavity in spherical harmonics

$$r=a[1-\epsilon \sum_{l=0}^{\infty }\sum_{m=-l}^{l}c_{lm}Y_{lm}(\theta ,\phi )],$$ (8)

and he provided explicit formulae for the splitting of the non-radial acoustic modes and for the second perturbations to the radial modes in terms of the expansion coefficients *c_lm_*. Comparable results for the splitting of microwave triplets have also been derived [6]. The splittings of the *l* = 1 microwave and acoustic modes depend on *ϵc*_20_ only, and by far the largest contribution to *ϵc*_20_ comes from the cylindrical extensions. Here we compare the results of the present dimensional measurements with the splittings measured near 20 °C.

**Table:** 

Source	10^4^*ϵc*_20_
P1997 dimensions	6.35
F1997 dimensions	5.40
(1,3) acoustic mode	5.48
(1,8) acoustic mode	5.63
TM11 microwave mode	5.61
TM12 microwave mode	5.56
TM13 microwave mode	5.51

Evidently, the observed splittings are more nearly consistent with the F1997 dimensions than the P1997 dimensions. The agreement of these subtle features of the resonator with theory is encouraging.

## 3. Microwave Measurements

Microwave measurements were used to determine the volumetric thermal expansion of the spherical cavity in the temperature interval 213 K to 303 K. Measurements were carried out in the vicinity of the TM11, TM12, and TM13 microwave triplets. Theory shows that the average frequency of these triplets was insensitive to geometric imperfections that leave the internal volume unchanged [5]. In addition, the accuracy of microwave measurements was demonstrated by directly comparing microwave measurements with mercury dilatometry [6].

### 3.1 Apparatus and Data Acquisition

For the measurements of *f*_m_, the acoustic transducer assemblies were removed from their ports and replaced with plugs from which the microwave coupling probes extended, exactly as described in [7]. These plugs had the same external dimensions as the acoustic transducer assemblies; thus, the ends of the plugs were recessed 0.9 mm from the interior surface of the resonator. For most of the present measurements, the coupling probes protruded 4.07 mm beyond the ends of the plugs. After most of the data were acquired, a few measurements were made with the probes shortened to 2.31 mm and a few measurements were made with the probes extended to as much as 20 mm. The effects of changing the length of the probes are discussed below.

During the microwave measurements, the interior of the resonator was evacuated while the surrounding pressure vessel was filled with helium to a pressure near 10 kPa to facilitate thermal equilibration. The valve atop the resonator was left open.

The temperature of the resonator was determined from two capsule-type platinum resistance thermometers that were embedded in metal blocks fastened to the top (thermometer #LN303) and bottom (thermometer #LN1888002) of the resonator. The temperature assigned to the resonator was always the average of the two thermometers. The thermometer’s resistances were recorded after each frequency measurement so that each mode had its own unique determination of the average temperature. Immediately after completion of the microwave measurements, the resistances of the thermometers were checked in a triple point of water cell. The values of *R*(*T*_w_) and the coefficients given in Table 6 were used to calculate the temperatures for the microwave data listed in Table A1. Although the intervals between complete recalibrations of these thermometers were quite long, the thermometers have a long history of good stability (see Table 5). We conservatively estimate that the uncertainty of the resonator’s temperature during the microwave measurements was no more than 2 mK relative to ITS-90 and this uncertainty propagates into relative standard uncertainties of [*a*(*T*)/*a*(*T*_w_)]^2^ and (*T*−*T*_90_) that are less than 0.1 × 10^−6^ because (1/*a*) d*a*/d*T* is only about 16 × 10^−6^ K^−1^.

The microwaves were generated and detected by a Hewlett-Packard Model 8753B network analyzer which was connected to the resonator through a Hewlett-Packard Model 85047A S-Parameter Test Set. The analyzer was configured to measure **S**_12_, which is defined (for properly terminated lines) as the complex ratio of voltage transmitted through the resonator to the voltage incident on the resonator. These instruments permitted measurements up to 6 GHz. We made detailed measurements in the vicinity of the nearly degenerate TM11, TM12, and TM13 triplets which occurred at 1.47 GHz, 3.28 GHz, and 5.00 GHz, respectively. The triply degenerate TE multiplets could not be detected with the straight probes that we used, and we decided to avoid the complications of dealing with multiplets with more than three components. In Ref. [6] it was shown that the thermal expansion determined from such highly degenerate multiplets is consistent with that obtained from the TM11 and TM12 triplets.

The frequency of the oscillator in the network analyzer was derived from the highly stable quartz reference oscillator installed in the audio frequency synthesizer used for the acoustic measurements. The frequency stability of this quartz oscillator was periodically confirmed by comparison with primary standards.

The network analyzer was used to scan 101 frequencies spanning each multiplet under study. The scan widths were 1.2 MHz, 1.6 MHz, and 2.0 MHz for the TM11, TM12, and TM13 triplets, respectively. Typically 20 scans with an IF bandwidth of 10 Hz were averaged. For each frequency, the averaged values of the real (*u*) and the imaginary (*v*) parts of the signal transmitted through the resonator were down-loaded to a computer for fitting. The values of *u* and *v* at all of the 101 frequencies were fit to a sum of two Lorentzian functions of the frequency,

$$u+iv=\sum_{m=1}^{2}\frac{ifA_{m}}{\left(f^{2}-F_{m}^{2}\right)}+B+C(f-f_{1}).$$ (9)

Here, ***A***, ***B***, and ***C*** are complex constants, and ***F*_m_** = *f*_m_ + *ig*_m_ are the complex, nearly-degenerate resonance frequencies of the triplet under study. In Eq. (9), the parameters ***B*** and ***C*** account for possible crosstalk and for the effects of the “tails” of the modes other than the one under study. Although the multiplets we studied were expected to be nearly degenerate triplets, the data could be represented by Eq. (9) summing over just two complex resonance frequencies (see Fig. 3). Typically the standard deviation of the fit, expressed as a fraction of the maximum amplitude measured, was 0.00016, 0.00035, and 0.00075 for the TM11, TM12, and TM13 triplets, respectively. Thus, the present signal-to-noise ratio was at least a factor of 4 larger than that achieved in Ref. [6]. The repeatability of the fitted frequencies and half-widths were always less than 100 Hz (or equivalently, (0.02 to 0.07) × 10^−6^ of the resonance frequencies). The frequencies and half-widths resulting from the fits to the microwave data are in Table A1 in the Appendix.

For the TM11 and TM13 triplets, the deviations from the fitted functions were random. This was confirmed by further averaging. The deviations were reduced by a factor of 3 and remained random. For the TM11 triplet, ***B*** and ***C*** in Eq. (9) were negligible. For the TM12 triplet, the deviations from the fitted functions were systematic and the parameter ***B*** in Eq. (9) accounted for nearly one quarter of the peak signal. These observations are a consequence of the proximity of the TM41 multiplet to the TM12 triplet and the comparatively efficient coupling of the TM41 multiplet to the probes. (The peak amplitude in the vicinity of the TM41 multiplet was 30 times the peak amplitude in the vicinity of the TM12.) Thus, it is not surprising that we were unable to improve the quality of the fit to the TM12 triplet by adding a third resonance frequency within the scanned range with a half-width comparable to the other two.

### 3.2 Interpretation of Microwave Frequency Measurements

#### 3.2.1 Partial Splitting of the Triplets

**Fig. 4:**
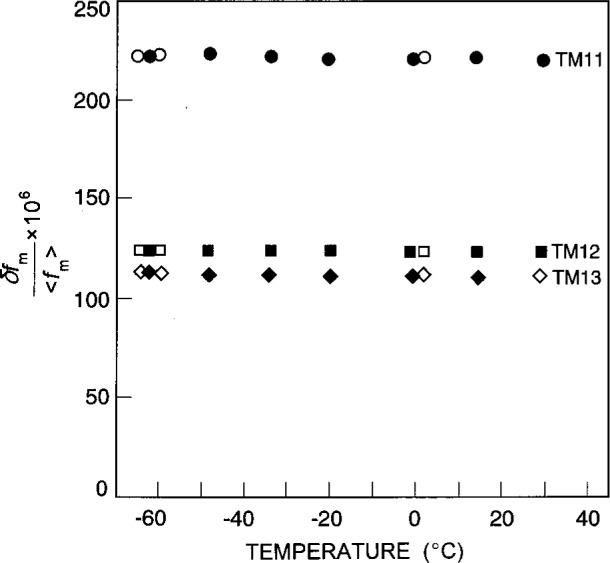
The temperature dependence of the frequency differences between the two resolved components of three microwave triplets, expressed as a fraction of the average frequency of each triplet. The open symbols denote data taken with shortened probes.

In Refs. [6,7], as in this work, the TM11 and TM12 triplets were fitted by just two resonance frequencies. In Ref. [6], it was argued that the third component of the expected triplet was not detected because the deviations of the cavity from a perfect sphere were primarily axisymmetric. Furthermore, it was argued from acoustic phase measurements that the lower frequency components of the TM11 and TM12 triplets were unresolved doublets and the upper components were singlets. We obtained further evidence supporting this conclusion by soldering a 20 mm long curved wire to one of the probes. This extension produced a partial splitting of all three components of the triplets. A multi-parameter fit to the TM13 triplet resulted in a splitting of 250 kHz between the lowest two components and a splitting of 602 kHz between the highest two components. The latter is close to the 660 kHz splitting between the doublet and the singlet that was observed with short probes. The fractional splittings [δ*f*/〈*f*_m_〉 in Eq. (10)] range from 111 × 10^−6^ to 222 × 10^−6^ (see Fig. 4).

$$\delta f/\langle f_{m}\rangle \equiv (f_{\text{singlet}}-f_{\text{doublet}})/(\frac{1}{3}\;f_{\text{singlet}}+\frac{2}{3}\;f_{\text{doublet}}).$$ (10)

**Fig. 5:**
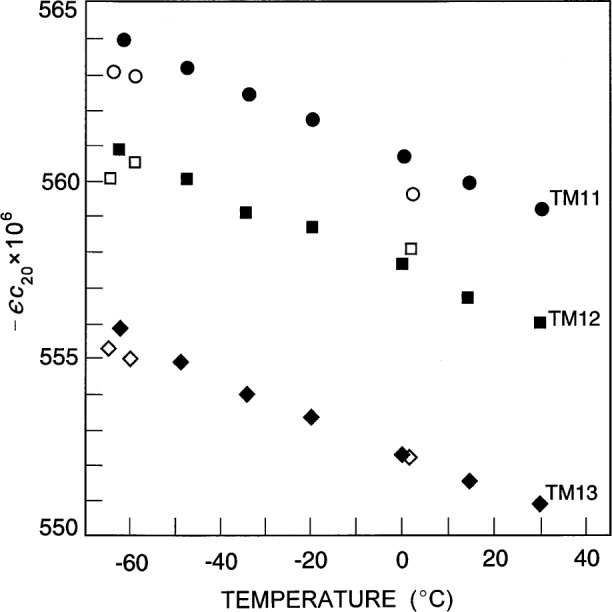
The temperature dependence of the deformation parameter −*ϵc*_20_ calculated by substituting the data of Fig. 4 into Eq. (12). Note that the zero of the ordinate is suppressed and that the data for the three modes are nearly consistent. This is evidence that the microwave splittings are mostly the result of a simple shape imperfection. The open symbols denote data taken with shortened probes.

Following Ref. [6] we note that if the splitting resulted from an axisymmetric deformation of the spherical cavity’s radius *a* such that the radial coordinate *r* was given by

$$r=a[1-\epsilon \sum_{l=0}^{\infty }c_{l0}Y_{l0}(\theta )],$$ (11)

the splittings of the *l* = 1 TM triplets would be

$$\frac{\delta f}{\langle f_{m}\rangle }=\epsilon c_{20}\left[-\frac{1}{2}-\frac{3}{{\left(v_{m}\right)}^{2}-2}\right]\frac{3}{\sqrt{20\pi }}.$$ (12)

(*v*_m_ is a microwave eigenvalue.) We have ignored any possible deformations with *l* > 2 and used the measured splittings together with Eq. (12) to determine *ϵc*_20_. For the three microwave triplets we found *ϵc*_20_ ≅ − 560 × 10^−6^. Remarkably, the values of *ϵc*_20_ differ from their mean by less than 1 % at each temperature. (See Fig. 5.) The negative values of *ϵc*_20_ imply that the polar “radius” of the resonator fractionally exceeds the equatorial “radius” by approximately 560 × 10^−6^. The temperature dependence of *ϵc*_20_ indicates that the thermal expansion of the resonator is not isotropic. As the temperature is increased from 213 K to 303 K the average radius of the cavity increases fractionally by approximately 1.4 × 10^−3^. However, the fractional increase of the polar radius is 5 × 10^−6^ less than that of the equatorial radius.

The open symbols in Fig. 5 indicate the values of *ϵc*_20_ obtained when the length of the probes was reduced from 4.07 mm to 2.31 mm. It is apparent that the probes had minor but detectable influence on the observed splitting of the TM11 mode.

#### 3.2.2 Widths of Microwave Resonances

The penetration of the microwave field into the wall of the resonator results in a contribution to the half-widths of the resonances *g*_m_ as well as an equal reduction in the frequencies of the resonances. The magnitude of these effects for the TM*lm* modes considered here is:

$$\frac{\Delta f_{m}+ig_{m}}{f_{m}}=(-1+i)\frac{\delta_{m}}{2a}{\left[1-\frac{2}{v_{m}^{2}}\right]}^{-1}.$$ (13)

In Eq. (13), *δ*_m_ is the electromagnetic field penetration length given by

$$\delta_{m}=\sqrt{\pi f_{m}\mu \sigma },$$ (14)

in which *σ* is the conductivity of the stainless-steel shell and *μ* is the magnetic permeability.

In order to calculate *g*_m_, we assumed that the microwave permeability of the cavity’s wall was exactly *μ*_0_, the permeability of free space, and that the electrical conductivity of the cavity’s wall at microwave frequencies was identical with the dc conductivity. The latter was estimated from the data of Clark et al. [18] who measured the resistivity of samples of 316 stainless-steels from four different manufacturers at five temperatures: 273 K, 192.4 K, 75.75 K, 19.65 K, and 4.0 K. For the three highest temperatures, the resistivity of each sample is very nearly a linear function of temperature. We represented the resistivity data in the range 213 K to 303 K by the following linear function of temperature *T*:

$$\rho /(\Omega \cdot m)=7.552\times 10^{-7}[1+1.26\times 10^{-3}(T-T_{0})],$$ (15)

where *T*_0_ ≡ 273.15 K. At 273 K resistivities of four samples ranged ±4 % about the value returned by Eq. (15). The corresponding range in calculated electromagnetic penetration lengths is 62 %. Using Eqs. (13) to (15), we calculated *g*_m_(calc.)/*f*_m_ = (87.2 ± 1.7) × 10^−6^ for the TM11 triplet and *g*_m_(calc.)/*f*_m_ = (29.8 ± 0.6) × 10^−6^ for the TM13 triplet. The uncertainty in *g*_m_(calc.) propagates into an uncertainty of 0.39 × 10^−6^ in [*a*(*T*)/*a*(*T*_W_)]^2^ at the lowest temperature of this study and 0.18 × 10^−6^ to the uncertainty in [*a*(*T*_g_)/*a*(*T*_W_)]^2^.

**Fig. 6:**
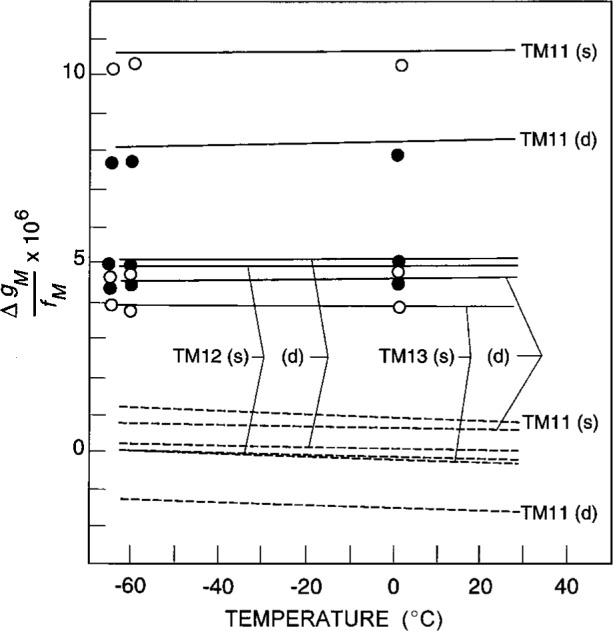
Temperatures dependence of the fractional excess half-widths of the single (s) and doublet (d) components of three microwave triplets [Δ*g*_m_ ≡ *g*_m_(meas.) − *g*_m_(calc.)]. The open circles represent the data taken with the shortened probes. The dashed curves show the same data recalculated with the assumption that the electromagnetic field penetration length is increased by 10 % over that calculated from the low-frequency resistivity.

Figure 6 compares the experimental values of *g*_m_ to the calculated values of *g*_m_. The plot shows the scaled excess half-width Δ*g*_m_ defined by

$$\Delta g_{m}/f_{m}\equiv [g_{m}(\text{expt}.)-g_{m}(\text{calc}.)]/f_{m}.$$ (16)

The values of Δ*g*_m_/*f*_m_ are positive, nearly temperature-independent, and differ for each component of each triplet. (In Fig. 6, the higher frequency components are labeled *s* and the lower frequency components are labeled *d*.) If Δ*g*_m_/*f*_m_ had a temperature dependence as strong as that of *g*_m_(expt.)/*f*_m_ it would have been detected.

Figure 6 shows that the effect of reducing the length of the coupling probes from 4.07 mm to 2.31 mm was detectable, but small. [Compare the solid circles (long probes) with the open circles (short probes).] Thus, the probes cannot explain most of Δ*g*_m_(expt.)/*f*_m_.

We noted that Δ*g*/*f*_m_ varied approximately as (*f*_m_)^−1/2^ This suggested that the phenomenon responsible for the excess half-widths was an evanescent wave and led us to reconsider the half-width data obtained with the shortened probes. We made the *ad hoc* assumption that the electromagnetic field penetration lengths were 10 % longer than those calculated above. This would be the case if the resistivity of our shell were 20 % larger than that measured for comparable alloys by Clark et al. [18] or if the magnetic permeability of our shell were 20 % larger than *μ*_0_. (The relative magnetic permeability of type 316 stainless steel is reported as 1.008. Cold working increases the permeability of type 316 slightly and greatly increases the permeability of similar alloys with slightly lower nickel content [19].) Using the larger penetration lengths, we found that Δ*g*_m_/*f*_m_ for all of the microwave components fell in a narrow range spanning zero (see the dashed curves on Fig. 6). With this same *ad hoc* assumption, the volumes determined from the three triplets span a range of only 4 × 10^−6^, fractionally. This contrasts with the range of 18 × 10^−6^ deduced from the smaller penetration lengths. The consequences of this alternative analysis on *T*/*T*_w_ were calculated and the difference between the two analyses contributed to the uncertainty of *T*/*T*_w_ that appears in Row 3 of Table 2.

### 3.3 Volumetric Thermal Expansion of the Resonator

To determine the thermal expansion of the resonator, we computed a “corrected” weighted average radius 〈*a*_m_〉 for each triplet at each temperature. Because the lower frequency component of each triplet is an unresolved doublet, we defined the average by

$$\langle a_{m}\rangle \equiv \frac{cv_{m}}{2\pi \left[\frac{2}{3}\;f_{\text{doublet}}+\frac{1}{3}\;f_{\text{singlet}}+g_{m}(\text{calc}.)\right]},$$ (17)

in which *g*_m_(calc.) is the value of the half-width computed from Eqs. (13), (14) and (15). With the probes at their “normal” 4.07 mm length, the values of 〈*a*_m_〉 for the TM11 triplet were always about 10 × 10^−6^ larger than the values of 〈*a*_m_〉 for the other triplets. (When the probes were shortened, this discrepancy was greatly reduced.)

**Fig. 7:**
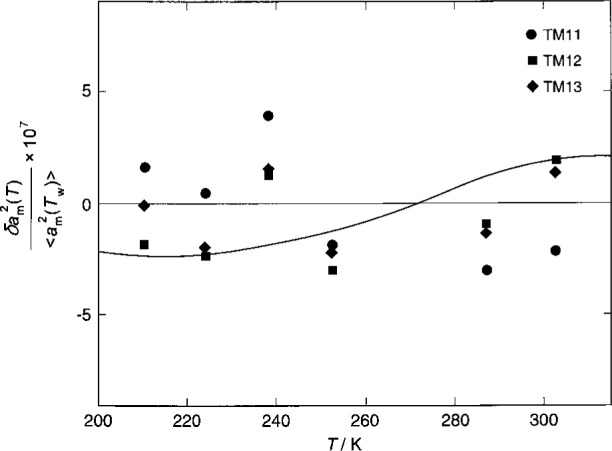
Fractional deviations of the microwave data from the temperature-dependent average internal radius 〈*a*_m_〉 of the resonator. The average radius for each TM1*m* microwave triplet is defined by Eq. (17) with the parameters of Eq. (18). The solid curve shows the small effect of replacing the theoretical values of the half-widths in Eq. (17) with their experimental values.

The microwave frequencies were measured during the 1989 runs and spanned the temperature range 210.74 K to 302.93 K. Because the microwave frequencies had not been measured near the 217 K isotherms used for the 1992 acoustic measurements, an interpolation function for the thermal expansion was needed. We fitted a polynomial function of (*T*_w_−*T*) to the three triplets of 〈*a*_m_(*T*)〉. The results can be represented by

$$10^{6}\left[{\left(\frac{\langle a_{m}(T)\rangle }{\langle a_{m}(T_{w})\rangle }\right)}^{2}-1\right]=-31.314437\bar{t}+0.01704{\bar{t}}^{2}+0.000041{\bar{t}}^{3},$$ (18)

with $\bar{t}\equiv (T_{w}-T)/K$. All the parameters in Eq. (18) had a significance greater than 0.999 based on the *F*-test and the Student *t*-distribution. The fractional standard deviation of the fit for [〈*a*(*T*)〉/〈(*T*_w_)〉]^2^ was 0.22 × 10^−6^. The deviations are shown in Fig 7. Clearly, the measurements of the microwave frequencies define a thermal expansion function with extraordinarily high precision. The curved line in Fig. 7 shows the effect of replacing the calculated halfwidths g_m_(calc.) in Eq. (17) with *g*_m_(expt.), the experimental values. The effect of this change is slight. The choice of the functional form for Eq. (18) is not critical. Indeed, if linear interpolation between adjacent microwave isotherms 210 K and 224 K had been used to obtain [〈*a*(*T*)〉/〈*a*(*T*_w_)〉]^2^ on the acoustic isotherm 217 K, the result would have differed by only 0.48 × 10^−6^ from Eq. (18), and this is the worst case.

We considered several alternatives to defining the radius by Eq. (17) and fitting 〈*a*_m_(*T*)〉 by Eq. (18). In reviewing the alternatives, recall that increasing [*a*(*T*)/*a*(*T*_w_)]^2^ by 1 × 10^−6^ increases *T*/*T*_w_ by 1 × 10^−6^ or, equivalently, 0.30 mK at *T*_g_. If we had used the measured half-width of each component of the multiplet instead of the calculated half-width in Eq. (17), [*a*(*T*)/*a*(*T*_w_)]^2^ would have changed by 0.24 × 10^−6^ in the worst case. If we had not deduced that the lower component of each triplet was an unresolved doublet and used the definition

$$\langle a_{m}\rangle \equiv \frac{cv_{m}}{2\pi \left[\frac{1}{2}\;f_{\text{doublet}}+\frac{1}{2}\;f_{\text{singlet}}+g_{m}(\text{calc}.)\right]},$$ (19)

the change in [*a*(*T*)/*a*(*T*_w_)]^2^ would have been less than 0.06 × 10^−6^. If we fit the data for each multiplet separately, the range of the values of [*a*(*T*)/*a*(*T*_w_)]^2^ would have been 0.4 × 10^−6^. The small effects of these changes is reassuring.

### 3.4 Comparison with Earlier Thermal Expansion Measurements

The present measurements of the volumetric thermal expansion are compared to earlier microwave measurements and to mercury dilatometry in Table 4. Using the weighted average of the TM1*m* modes we find 10^6^[*V*_0_(*T*_g_)/*V*_0_(*T*_w_) − 1] = 1419.0 ± 0.7. The discrepancy with the previous measurements is displayed in Table 4 and is equivalent to 0.25 × 10^−6^ in *V*_0_(*T*_g_)/*V*_0_(*T*_w_), when the ratio from Ref. [6] is recalculated using the values of *g*_m_ obtained from Eqs. (13) to (15). (In Ref. [6] the expression used for the resistivity differed slightly from Eq. (15).) The two sets of microwave measurements agree to well within estimated uncertainties. The fractional difference between this determination and the mercury dilatometry is 2.45 × 10^−6^; this is within 1.5 combined standard uncertainties.

## 4. Acoustic Measurements

With the minor exceptions mentioned below, the complex resonance frequencies ***F***_a_ ≡ *f*_a_ + *ig*_a_ of the radially symmetric, non-degenerate acoustic modes were determined with the same transducers, instruments, and procedures that were used with this resonator in the past [3,4,7]. For brevity, we describe the exceptions and the changes while omitting repetition of details that are unchanged.

For argon, the frequencies and half-widths of the lowest five radial modes [conventionally designated (0,2), (0,3), … (0,6)] were measured. As discussed below, the (0,6) mode had a small, anomalous pressure dependence that may have resulted from a near coincidence of the frequency of that mode with a mode of the spherical shell. To explore the effect of this, a separate analysis was conducted omitting all the argon data for the (0,6) mode.

For xenon, the frequencies of lowest seven acoustic modes were measured; however, only the lowest five were used in the final analysis. We knew (see Fig. 8 of Ref. [4]) that the resonance frequencies of the (0,7) and (0,8) modes partially overlap those of nearby, highly degenerate, non-radial modes; however, we expected that the effects of the overlap could be canceled to a high degree when computing speed-of-sound ratios on a mode by mode basis, as implied by Eq. (7). However, the uncertainties of the xenon data were dominated by unanticipated impurity effects. This, and the fact that the mode-by-mode analysis proved more cumbersome than anticipated led us to discard the data for the (0,7) and (0,8) modes.

In Ref. [4], arcing within the detector transducer was reported. On occasion, the problem of arcing within the detector transducer reappeared. It was permanently cured by reducing the dc bias voltage on the detector transducer from 200 V to 150 V. The reduction of the bias voltage reduced the signal-to-noise ratio approximately 25 %. This was more than offset by altering the protocol for measuring the signal produced by the detector transducer. In our earlier work, an analog lock-in amplifier had been used to measure the detected acoustic signal and the amplifier’s post-detection filter was used for averaging. Then, one had to wait eight filter time constants for the output to settle fractionally to within 3 × 10^−4^ of its final value before recording the output, and the recorded output benefitted from one-filter-time-constant of averaging. In this work, we used a digital lock-in amplifier. The post-detection time constant of this amplifier was set to 0.3 s. After each increment of the acoustic frequency, we waited for the output of the lock-in amplifier to settle to within 10^−4^ of its final value. (The settling occurred with the time constant *τ*_s_ ≡ 1/(2π*g*_a_).) Then, the output of the lock-in amplifier was measured at intervals of *τ*_s_ and digitally averaged. In comparison with our previous work with an analog lock-in amplifier, the measurement time was unchanged and the signal-to-noise ratio was increased up to a factor of 3 as the pressure was reduced below 100 kPa. (In previous work [4] below 100 kPa, *τ*_s_ < 1 s, the post-detection filter had been set to 3 s, and the signal-to-noise ratio decreased as *p*^−2^.)

For both argon and xenon, each complex acoustic resonance frequency ***F****_a_* was determined by fitting the detector’s response to a single Lorentzian function of the frequency (Eq. (9) with the summation index *m* taking on the value 1, only). Equation (9) was fitted to the data for each mode twice, once with the constraint ***C*** ≡ 0, and once without the constraint. The constrained fit was used for further analysis, except when relaxing the constraint reduced the standard deviation of the fit by at least 30 %.

For weighting fits of the temperature and pressure dependencies of *f*_a_, it is convenient to have an approximate expression for the standard deviation of a single measurement of *f*_a_. An expression for argon was developed during the redetermination of *R* and of *T*_g_:

$$\sigma (f_{a})=10^{-7}f_{a}[1+{\left(100\mathrm{kPa}/p\right)}^{2}{\left(6\;\mathrm{kHz}/f_{a}\right)}^{2}].$$ (20)

At pressures above 100 kPa the signal-to-noise ratio was sufficiently high that the imprecision of a measurement was dominated by small, uncontrolled phase shifts in the measurement system. The loss of precision at low pressures is a consequence of the signal declining as *p*^3/2^ and the resonance half-widths increasing as *p*^−1/2^. When the resonator was filled with xenon the transducers’ characteristics were essentially unchanged. For xenon, Eq. (20) is a reasonable estimate for *σ* (*f*_a_), provided that the characteristic pressure in that equation is replaced with 55 kPa, in accordance with expectations based on xenon’s thermophysical properties. In this work, we continued to use Eq. (20) with the characteristic pressures 100 kPa and 55 kPa to weight the data, even though Eq. (20) over-estimated the uncertainty of the lowest-pressure data.

The systematic errors arising from the reference oscillator in the frequency synthesizer, non-linear effects, and the instrumentation for frequency measurement were found to be negligible.

Prior to the commencement of the acoustic measurements, the resonator was evacuated and “baked” at 50 °C for 4 d. Simultaneously, the gas manifold was baked at or above 100 °C. At the conclusion of the baking procedure the residual pressure indicated by an ionization gauge was approximately 13 μPa. After the acoustic measurements at *T*_w_, *T*_m_, and 217 K, the resonator was again baked, this time at 60 °C for 24 h. After the conclusion of the argon measurements and prior to the xenon measurements at *T*_w_, the gas manifold was baked again at 100 °C for 24 h to remove any residual traces of argon from the pipework. The resonator was baked again before the final xenon measurements at *T*_g_.

At *T*_w_, the acoustic measurements were made at 13 pressures between 25 kPa and 500 kPa in three separate fillings of the resonator. On the other isotherms, the measurements were made at pressures which corresponded to approximately the same densities. (Only at 217 K, where the O-ring sealing the pressure-vessel leaked, was it necessary to limit the maximum working pressure.) In this way, we ensured that the number of parameters required to fit the virial expansion to the acoustic data was the same on every isotherm studied and we avoided the possibility of biasing *T*/*T*_w_ by using different orders of fit on various isotherms. In xenon, measurements were conducted at pressures in the range 30 kPa to 300 kPa.

For both argon and xenon, the frequencies and half-widths of the radially symmetric modes were determined twice at each temperature and pressure, first in order of ascending mode index, and then in the reverse order. The resistances indicated by the three thermometers were recorded after each frequency measurement, ensuring that each mode had a unique determination of the mean temperature of the cavity.

## 5. Thermometry

### 5.1 Temperature of the Bath

A calibrated thermometer was used to measure the temperature of a point within the bath. This facilitated adjusting the bath’s temperature to within a few mK of the resonator’s temperature. Because the bath’s temperature was so close to the resonator’s temperature, the latter usually drifted less than 0.5 mK during the 45 min required to acquire the acoustic data at each temperature and pressure.

### 5.2 Temperature of the Resonator

The temperature of the gas within the cavity was inferred from three capsule-type standard platinum resistance thermometers (SPRTs). Two of these thermometers (LN1888002 and LN303) had been used during the redetermination of *R* and of *T*_g_. These were mounted in metal blocks attached to the bosses at the top (LN303) and bottom (LN1888002) of the resonator. The third SPRT (Chino serial number RS18A-5) was purchased after the microwave measurements were completed and installed near the equator of the resonator prior to the acoustic measurements of 1989. This thermometer was surrounded by two metal sleeves that were threaded into the resonator. The inner sleeve was OFHC copper and the outer was aluminum. The resistances of each thermometer were measured before and after each measurement of *f*_s_ and the temperature associated with that measurement was the average of the temperatures of the three thermometers.

### 5.3 Thermometer Calibration, History and Stability

We now describe the other factors which lead to the uncertainty estimates in Table 2 under the heading “Thermometry”. The Aresistance bridge, its standard resistors, and the SPRTs together function as a transfer and interpolation standard between the triple point cells and the resonator. Therefore, the primary concern is the long term stability of the thermometers. This was evaluated by periodically checking the thermometers in triple point cells. Table 5 lists the quantity *R*(*T*_t_, *i* → 0) which is the resistance measured with the 30 Hz ac bridge extrapolated to zero current. Here, *T*_t_ represents the temperature of the triple point t where the subscript “t” stands for “w”, “m”, “g”, or “a” in the notation *T*_w_, *T*_m_, *T*_g_, or *T*_a_ which denote the temperatures of the triple points of water, mercury, gallium, or argon, respectively. (In contrast, Refs. [4] and [7] use the symbol *T*_t_ to represent the temperature of the triple point of water.) The average change in *R*(*T*_t_, *i* → 0) between our calibrations is 10 μΩ. We used this value as the estimated standard uncertainty in (*T*−*T*_90_)/*T* resulting from our imperfect thermometer calibrations and listed the results in Row 8 of Table 2.

The calibrations at *T*_w_, *T*_m_, and *T*_g_ just before (May 1992) and just after (August 1992) the acoustic measurements, together with subsequent (October 1992) calibrations at *T*_w_ and *T*_a_ were used to generate two sets of coefficients for the defining equations on ITS-90. The two sets of coefficients were used to calculate two temperatures (*T*_before_ and *T*_after_) for each isotherm. The temperature differences (*T*_before_−*T*_after_) are one measure of the uncertainty of *T* resulting from imperfect calibrations. The scaled differences 10^6^ × (*T*_before_−*T*_after_)/*T* are listed in Row 9 of Table 2. These differences arise from a common source; thus, they are not random. Indeed, they are highly correlated.

Table 6 lists values for the parameters *a* and *b* which occur in the definition of the International Temperature Scale of 1990. These values were determined from the calibrations before the acoustic measurements, given in Table 5, in conjunction with the triple point of argon measurements performed by the NIST Thermometry Group after the completion of the acoustic measurements.

To determine temperatures from the experimental resistance ratios, *W*(*T*_90_), the specified deviation functions for the appropriate ranges were used in conjunction with the parameters given in Table 6 to determine the reference function *W*_r_(*T*_90_). The specified inverse function was then used to calculate approximate temperatures on *T*_90_. The derivative of this function with respect to *W*_r_(*T*_90_) was determined numerically and used to adjust the *T*_90_ temperatures such that *W*_r_(*T*_90_) calculated from the deviation function and the reference function in conjunction with the assigned *T*_90_ value agreed to better than 10^−9^. This ensured that the *T*_90_ temperatures determined from *W*_r_(*T*_90_) could be calculated with arbitrary precision for a given resistance ratio *W*(*T*_90_). In this way, any error in the calculated temperatures resulted entirely from errors in the calibrations, sub-range inconsistencies, and non-uniqueness of the scale. The first of these errors is estimated from repeated calibrations at the various triple points, and the last two are inherent limitations of the scale for thermometers having equivalent calibrations. Above, we have specified the sub-ranges that we have used and have indicated them in Table 6. Thus the sub-range inconsistences do not contribute to the uncertainties of (*T*−*T*_90_) if “*T*_90_” is understood to mean the specified sub-range.

The non-uniqueness of ITS-90 does not contribute to the uncertainty of our values for either *T*_g_/*T*_w_ or *T*_m_/*T*_w_; however, it does contribute to the uncertainties of (*T*−*T*_90_) on our isotherms at 217 K, 253 K, and 293 K. From Fig. 1.5 of Ref. [20], we estimate the non-uniqueness of ITS-90 is 0.2 mK at 217 K and 253 K, provided the thermometers are calibrated at *T*_w_, *T*_m_, and *T*_a_. Because of the dearth of relevant published data, we made two naive observations to estimate the non-uniqueness contribution to the uncertainty of (*T*−*T*_90_) near 293 K. First, we noticed that the non-uniqueness varies approximately parabolically over the interval between closely spaced calibration points, and second, the interval (*T*_g_−*T*_w_) is approximately 0.77 × (*T*_w_−*T*_m_). Thus, we expect the contribution to be (0.77)^2^ times the 0.2 mK contribution at 253 K. The non-uniqueness contributions to the uncertainty of (*T*−*T*_90_)/*T* appear in Row 11 of Table 2.

### 5.4 Calibration Techniques

The resistances of the thermometers were measured with a four-wire, ac resistance bridge operated at 30 Hz. The bridge was designed by R. D. Cutkosky and built at NBS and has been designated NBS/CAPQ Microhm Meter 5. The bridge was used for all of the thermometer calibrations and all of the temperature measurements; thus, uncertainties associated with differences between bridges were not present [21]. This bridge is the same one which had been used for the re-determinations of *R* and *T*_g_ [4,7]. Normally, the bridge was operated with a measurement current of 1 mA and, with our 25 Ω thermometers installed in the resonator, a typical standard deviation of reading was 3μΩ.

For calibration, each capsule thermometer was installed in an extension probe similar to that described in Ref. [22]. Calibration resistance measurements were performed with currents of 1 mA and 2 mA so that we could extrapolate the results to zero current. The calibration measurements were always performed in the order 1 mA, 2 mA and 1 mA during a period of at least 10 minutes *after* the SPRT had equilibrated in the triple-point cells. When unexpected drifts occurred, they were observed and the cause was eliminated prior to repeating the measurement. In this way a standard deviation of a calibration measurement was 1μΩ. When installed in the resonator, the self-heating of the thermometers was within 10 μΩ·mA^−2^ of that measured when the thermometers were installed in the calibration probes.

The gallium point cell used for the calibrations was the same one that had been used in the redetermination of *T*_g_. As mentioned in Ref. [7], B.W. Mangum (then in the Temperature and Pressure Division of the NBS) compared this cell with one of a group which he maintained as standards. The two cells were indistinguishable at the level of 50 μK. For storage this cell was filled with argon; it was evacuated prior to use.

The mercury point cell was manufactured and filled by us for this project following the guidelines of Furukawa et al [23]. The mercury used in the cell was a portion of the sample that had been used to determine the volume of the resonator in connection with the redetermination of *R*. This mercury is traceable to A. H. Cook and came from the same NBS stock as described in Refs. [4] and [7]. All glass used in the construction of the cell was cleaned with HF prior to use.

In March 1998, G. F. Strouse of NIST’s Thermometry Group made two direct comparisons of our mercury triple-point cell (Hg 88-1) with the Thermometry Group’s laboratory standard mercury triple-point cell (Hg SS-1). The measurement system included an ASL Model F18 bridge operating at a frequency of 30 Hz with a 100 Ω Tinsley Model 5685 reference resistor and a 25.5 Ω SPRT. Corrections were made for the difference in hydrostatic head effects due to the different immersion depths. After corrections, the triple-point temperature of the Hg 88-1 cell was 50 μK lower than that of the Hg SS-1 cell. A standard uncertainty of 0.10 mK was attributed to the value of *T*_m_ realized in the Hg SS-1 cell to account for the impurities and measurement uncertainties.

The calibration at the *T*_a_ reported in Table 5 was made by G.F. Strouse of the Thermometry Group of NIST using the resistance bridge mentioned above.

Our realization of the metal triple points was by means of the “double-melt” method. This method was selected in preference to the freezing method because it circumvents the problem of the massive undercool often observed when performing a “double-freeze.”

### 5.5 Temperature Gradients in the Resonator

**Fig. 8:**
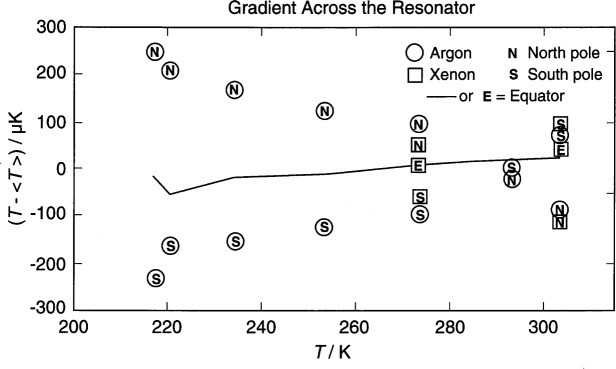
Differences between the temperatures of the thermometers and their mean (denoted 〈*T*〉). “N” denotes the thermometer at the top (or “north pole”) of the resonator and “S” denotes the thermometer at the bottom (or “south pole”). The temperature of the thermometer near the middle (or “equator”) of the resonator is indicated by the jagged line; however, the data taken when argon was in the resonator are *not* plotted on the line for clarity.

During the acoustic measurements a small vertical temperature gradient existed in the resonator. (See Fig. 8.) The gradient was nearly symmetrical about the equator. In the worst case, when the bath was at 217 K, the top of the resonator was 0.5 mK warmer than the bottom of the resonator. The gradient may have been caused either by an undetected heat leak from the laboratory to the top of the resonator or by temperature stratification within the stirred bath surrounding the pressure vessel. Either possible cause is consistent with the observation that as the temperature was increased from 217 K through room temperature to *T*_g_, the magnitude of the gradient decreased, reaching zero at room temperature, and then increased with reversed sign.

One can show that the acoustic resonance frequencies *f*_a_ for the radially symmetric modes are determined by the volume average of the temperature distribution within the gas. Furthermore, if the deviation of the temperature from the mean is represented by δ*T*(*r*,*Θ*,*ϕ*) the frequency shifts are of the order of 〈δ*T*^2^(*r*,*Θ*,*ϕ*)〉. Thus, only the asymmetry in the measured vertical gradient contributes to the uncertainty of *T*; this was probably less than 0.1 mK and therefore negligible.

We had no means of detecting a horizontal component to the temperature gradient. If such a gradient were present, it probably was smaller than the vertical gradient because the resonator, the cylindrical copper heat shield enclosing it, and the pressure vessel enclosing both were all axisymmetric (Fig. 2). Furthermore, the dimensions of the heat shield were such as to screen horizontal gradients more effectively than vertical gradients. Somewhat arbitrarily, we assumed that any asymmetry of the horizontal temperature gradient was one half the temperature difference between either end of the resonator and its middle. Row 10 of Table 2 lists the uncertainty in (*T*−*T*_90_)/*T* corresponding to ½ the temperature difference between the middle of the resonator and the top of the resonator.

As observed in Ref. [4], closing the isolation valve in the top of the resonator heated the thermometer attached to the top of the resonator 1 mK to 3 mK. Before measuring the acoustic frequencies, we waited until this thermal transient had decayed and the thermometers indicated temperatures that were identical with the ones before the valve was closed.

## 6. Gas Samples

The argon was withdrawn from the cylinder that contained the gas used in the re-determination of *R* and *T*_g_. This cylinder had been purchased from Matheson Gas Products. The supplier’s lot analysis provided the following upper bounds for the mole fractions of impurities: N_2_ < 3 × 10^−6^; O_2_ < 1 × 10^−6^; H_2_O < 1 × 10^−6^; and total hydrocarbons < 0.5 × 10^−6^. No further purification was attempted. The small effects of these impurities on the speed of sound in argon can be found in Ref. [4].

The xenon was “research-grade” purchased from Matheson Gas Products. The supplier’s lot analysis provided the following upper bounds for mole fraction of impurities: N_2_ < 2 × 10^−6^; O_2_ < 1 × 10^−6^; and Kr < 18 × 10^−6^. This sample was purified by exposing it for 91 h to a zirconium-aluminum alloy getter maintained at 673 K. Under these conditions, the getter is very effective at removing gases such as CO, CO_2_, O_2_, N_2_, and H_2_ from the noble gases and it is moderately effective at removing hydrocarbons. After purification, the xenon was condensed into a small stainless steel cylinder for storage prior to being admitted into the resonator. This storage cylinder had been baked under high vacuum at a temperature above 400 K until the residual pressure was less than 10 μPa (about 10^−7^ torr). The change of the speed of sound in xenon upon addition of an impurity with a mole fraction *x* << 1 can be calculated from the quantity (1/*u*^2^)(d*u*^2^/d*x*). For the impurities of interest, this quantity has the values: N_2_, 0.52; O_2_, 0.49; H_2_O, 0.43; CO_2_, 0.40; Ar, 0.70; and Kr, 0.36. The monatomic impurities that were not removed by the getter would not affect the ratios *T*/*T*_w_ unless their mole fractions changed (for example, by preferential adsorption) when the temperature changed. (See Sec. 8.4 for a detailed discussion of possible contamination.)

## 7. The Pressure and Other Thermophysical Properties

The present determinations of *T*/*T*_w_ require measurements of the pressure and values for the thermal diffusivity of the gases; however, these quantities need not be known with nearly the same accuracy as the primary quantities. Expressions are presented for the virial coefficients, thermal conductivities, and viscosities of argon and xenon.

### 7.1 Pressure Measurements

The pressure was measured with a fused-quartz, bourdon-tube, differential pressure gauge (Ruska Instrument Corporation, Model No. 6000-801-1). The gauge had a full-scale range of 1 MPa and had a resolution of 1 Pa. The reference side of the gauge was continually evacuated with a mechanical pump to a pressure lower than 1 Pa. At the conclusion of the measurements the gauge was calibrated against an air-lubricated pressure balance designated “PG29” by the NIST Pressure Group (Ruska Instruments, Model 2460). The pressure indications from the bourdon-tube gauge were up to a factor of 4.5 × 10^−4^ larger than the standard. The calibration data were represented by the equation

$$p/\mathrm{Pa}=R_{b}(1-4.556\times 10^{-4}),$$

with a standard deviation of 4 Pa. (Here, *R*_b_ is the numerical value of the pressure indicated by the quartz bourdon gauge.) Between checks, the largest change of the zero-pressure indication of the gauge was 35 Pa; the average change between checks was 15 Pa.

The gas in the resonator was separated from the manifold by a differential pressure transducer (DPT) constructed of stainless steel and Inconel (MKS Instruments model 315BD-00100 sensor head with model 270B electronic display unit). The DPT had a full-scale range of ± 13 kPa and a resolution of better than 1 Pa. At zero differential pressure, the DPT’s output voltage *V*_0_ varied with line pressure as

$$V_{0}/V=2.744\times 10^{-8}R_{b},$$

where *R*_b_ is the numerical value of the pressure indicated by the quartz bourdon gauge. This equation was applicable up to 600 kPa and fit the results with a standard deviation of 1.2 mV (corresponding to 1.6 Pa). Between checks, the largest drift in *V*_0_ was equivalent to 12 Pa; however the average drift was 5 Pa.

The DPT was calibrated over its full-scale range of ± 13 kPa at various line pressures between 0 kPa and 600 kPa before the acoustic measurements were made. The calibration depended upon both the sign of the differential pressure and the line pressure. The results for a positive differential pressure Δ_+_*p* were represented by the equations

$$\Delta_{+}p/\mathrm{Pa}=(V_{c}/V)[1.00213+2.299\times 10^{-14}{R_{b}}^{2}-2.469\times 10^{-7}(V_{c}/V)]$$

with a standard deviation of 7 Pa. For negative differential pressures Δ_−_*p*, we found

$$\Delta_{-}p/\mathrm{Pa}=(V_{c}/V)[0.99848+2.187\times 10^{-7}R_{b}+6.939\times 10^{-11}{\left(V_{c}/V\right)}^{2}]$$

with a standard deviation of 7 Pa. In these equations, *V*_c_ ≡ *V* − *V*_0_ is the voltage output of the capacitance differential pressure transducer corrected for the voltage output at zero pressure.

We estimate that the standard uncertainty of a single measurement of the pressure of the argon was approximately 19 Pa. For xenon, we checked the zero-pressure reading of the bourdon tube gauge after every pressure measurement. In this case, the standard uncertainty of a pressure reading was approximately 10 Pa.

In this work, (*T*−*T*_90_) was deduced by fitting polynomial functions of *p* to values of *u*^2^(*p*,*T*) and extrapolating to *u*^2^(0,*T*) along isotherms. The pressure-dependent contribution to the uncertainty of (*T*−*T*_90_) appears on Row 13 of Table 2. For each isotherm, the contribution was estimated by multiplying the uncertainty of the zero of the pressure gauge by the linear term in *u*^2^(*p*,*T*), i.e., *A*_1_ in Eq. (26). The pressure-dependent uncertainties are comparatively small.

### 7.2 Thermal Conductivity and Viscosity of Argon and Xenon

The thermal conductivity *κ* of the gas affects the determination of *T* through the thermal-boundary-layer term in the corrections to the acoustic frequencies Δ*f*_a_. Since the uncertainties in the values of *κ* at both *T* and *T*_w_ are correlated, the estimated 0.3 % relative standard uncertainty of *κ* contributes only 0.2 × 10^−6^ to the standard uncertainty in *T*/*T*_w_, as indicated on Row 12 of Table 2. The viscosity *η* of the gas contributes to the half-widths of the acoustic resonances and to the very small terms in Δ*f*_a_ resulting from crevices. Thus the uncertainty of the viscosity may be neglected when computing the standard uncertainty of *T*/*T*_w_.

We represented the thermal conductivity and viscosity of argon with the expression

$$\begin{array}{c} \kappa (T,\rho )/(\mathrm{mW}\cdot m^{-1}\cdot K^{-1})=6.4622\times 10^{-2}(T/K)\\ -7.7489\times 10^{-8}{\left(T/K\right)}^{3}+5.4288\times 10^{-11}{\left(T/K\right)}^{4}\\ +21.6\times 10^{-3}[\rho /(\mathrm{kg}\cdot m^{-3})] \end{array}$$ (21)

and

$$\begin{array}{c} \eta (T,\rho )/(\mu \mathrm{Pa}\cdot s)=8.2822\times 10^{-2}(T/K)-1.0088\\ \times 10^{-7}{\left(T/K\right)}^{3}+7.1206\times 10^{-11}{\left(T/K\right)}^{4}+11.10\\ \times 10^{-3}[\rho /(\mathrm{kg}\cdot m^{-3})] \end{array}$$ (22)

respectively. For Eqs. (21) and (22), the zero-density values of *κ* (*T*) and *η* (*T*) were calculated from the HFD-B2 potential by Aziz and Slaman [24]. (The HFD-B2 potential exploits theoretical results and simultaneously represents measurements of transport properties, density virial coefficients, spectral data for argon dimers, and molecular beam scattering.) As discussed by Aziz and Slaman and in Refs. [4] and [7], the transport properties derived from this potential are in agreement with recent measurements within experimental uncertainties, which range from 0.2 % to 0.3 %. The density coefficients of the thermal conductivity and viscosity are the values tabulated by Maitland et al. [25].

The corresponding expressions for xenon are

$$\begin{array}{c} \kappa (T,\rho )/(\mathrm{mW}\cdot m^{-1}\cdot K^{-1})=1.8986\times 10^{-2}(T/K)\\ -1.9528\times 10^{-6}{\left(T/K\right)}^{2}+6.0\times 10^{-3}[\rho /(\mathrm{kg}\cdot m^{-3})] \end{array}$$ (23)

and

$$\begin{array}{c} \eta (T,\rho )/(\mu \mathrm{Pa}\cdot s)=0.07952(T/K)-8.256\\ \times 10^{-6}(T/K{)}^{2}+6.17\times 10^{-3}[\rho /\mathrm{kg}\cdot m^{-3}] \end{array}$$ (24)

respectively. For Eqs. (23) and (24), the zero-density values of *κ* (*T*) and *η* (*T*) were calculated from the HFD-B2 potential of Dham et al. [26] by Aziz [27] and the density coefficients were obtained from Ref. [25].

### 7.3 Density Virial Coefficients

In order to correct the acoustic frequencies for the thermal boundary layer, we required the density *ρ* (*T*,*p*) and the constant-pressure molar heat capacity *C*_p_(*T*,*p*) of the gases as functions of the temperature and the pressure. To obtain *ρ* (*T*,*p*), we inverted the virial equation of state. We obtained *C*_p_(*T*,*p*) from thermodynamic relations that use the virial coefficients and their temperature derivatives.

For argon, the second virial coefficient *B*(*T*) and its temperature derivatives were calculated from the modified HFD-B2 potential function of Ewing and Trusler, correct to the second quantum correction [28]. The parameters for this potential were determined solely from their recent precise measurements of the second acoustic virial coefficient of argon over a wide range of temperatures. Because of the underlying data, we expect that this potential function will return estimates of the virial coefficients that are more accurate than those from the HFD-B2 potential of Aziz and Slaman [24]. The results were approximated by the polynomial

$$\begin{array}{c} B/({\mathrm{cm}}^{3}\cdot {\mathrm{mol}}^{-1})=34.236-1.166\times 10^{4}/(T/K)\\ -9.523\times 10^{5}/{\left(T/K\right)}^{2} \end{array}$$

with a negligible contribution to the uncertainties. Values of third virial coefficient *C* were taken from [29].

The virial coefficients of xenon and its temperature derivatives were determined from the experimental data of Michels et al. [30]. The expression resulting from a polynomial fit to the data was

$$\begin{array}{c} B/({\mathrm{cm}}^{3}\cdot {\mathrm{mol}}^{-1})=58.509-0.3681\times 10^{5}/(T/K)\\ -0.5532\times 10^{7}/{\left(T/K\right)}^{2}. \end{array}$$

Values of *C* were also taken from Ref. [30].

## 8. Analysis of the Acoustic Data

In this section we describe the analysis of the acoustic data. The resonance frequencies of the radial modes were corrected and then combined with values of *a*_0_(*T*), the radius at zero-pressure, that had been obtained from the microwave measurements. The resulting values of *u*(*p*,*T*)/*a*_0_(*T*) are listed in the Appendix in Table A2. The values of [*u*(*p*,*T*)/*a*_0_(*T*)]^2^ were fitted to a polynomial (acoustic virial) expansion in the pressure to obtain the zero-pressure limits [*u*_0_(*T*)/*a*_0_(*T*)]^2^. The fitting was done in two, quite different, ways. First, the data for each isotherm were fitted by independent polynomials, and second, all of the data were fitted simultaneously to a *u*^2^(*p*,*T*) surface. The results of both fitting procedures were used to compute the ratios [*u*_0_(*T*)/*a*_0_(*T*)]^2^/[*u*_0_(*T*_w_)/*a*_0_(*T*_w_)]^2^. To obtain *T*/*T*_w_, these ratios were combined with the radius-ratios determined from the microwave measurements and represented by Eq. (18). The temperature ratios were combined with the temperature measurements on ITS-90 to obtain (*T*−*T*_90_).

The remainder of this section is subdivided as follows: Sec. 8.1 describes the reduction of the raw data; Sec. 8.2 describes the fits of the individual argon isotherms; Sec. 8.3 describes the fitting of the argon *u*^2^(*p*,*T*) surface; Sec. 8.4 discusses variations of the fitting procedures, and Sec. 8.5 discusses the somewhat different analysis of the xenon data.

### 8.1 Reduction of the Acoustic Data to Speeds of Sound on Isotherms

The raw data for both argon and xenon were reduced to values of (*u*/*a*) on each isotherm in the following manner. The resonance frequencies were corrected for the thermal boundary layer, the effects of curvature of the surface, the temperature jump (assuming the thermal accommodation coefficient *h*=1), the coupling of gas and shell motions, and the effect of bulk dissipation. The resonance frequencies of the (0,2) mode were multiplied by the factor 1 + 0.7 × 10^−6^ to correct for the shape perturbation resulting from the unequal diameters of the hemispheres. All of these corrections are identical to those applied in previous work with this resonator and are given in Refs. [3] and [4]. As in Ref. [4], corrections were not applied for the small effects of the crevices surrounding the transducers because the geometry was not known well enough. The corrections for the static and dynamic compliances of the shell were taken from Ref. [2] and the dynamic compliance correction included the effect of radiation from the external surface. The elastic constants required for these corrections were taken from Ref. [4]; their temperature dependencies were taken from the measurements of Ledbetter et al. [31]. Finally, the frequencies were corrected to the exact isotherm temperatures *T*_i_ using

$$f(T_{i},p)=f(T,p){\left(T_{i}/T\right)}^{1/2}$$

with negligible additional uncertainty. The corrected frequencies were divided by the appropriate eigenvalue and by *a*_0_(*T*).

For tabulation only, the mean values of (*u*/*a*_0_) were computed for the first five radial modes. These means are listed in Table A2 of the Appendix along with pressures, temperatures, and the relative standard deviations of the mean.

For fitting, the corrected frequencies were divided by the appropriate eigenvalue and by *a*_0_. At each temperature and pressure there were two measurements made for each mode and the two values (*u*/*a*_0_) deduced from them were averaged. However, the values of (*u*/*a*_0_) for the five modes were *not* averaged so that the residual mode-dependence of the data could be studied. Following Refs. [4] and [7], each value of (*u*/*a*_0_) was weighted inversely by the square of its estimated standard deviation which was taken to be ${\left(\sigma_{1}^{2}+\sigma_{2}^{2}\right)}^{1/2}$. The first term *σ*_1_ is the estimate of the standard deviation of the frequency measurements from Ref. [7]:

$$\sigma_{1}=1.4\times 10^{-7}u^{2}[1+{\left(10^{5}\mathrm{Pa}/p\right)}^{2}{\left(6\;\mathrm{kHz}/f_{0n}\right)}^{2}].$$ (25)

The second term, *σ*_2_ = 3.7 × 10^−7^*u*^2^, accounts for the uncertainties of the mode-dependence of the model for the entire set of measurements. This term was chosen such that *χ*^2^ = 1 for a “good” fit, as judged by deviation plots. The fitting procedures were adapted from Bevington [32].

### 8.2 Fitting Independent Acoustic Isotherms: Argon

The corrected values of (*u*/*a*_0_)^2^ were fitted by the expansion in powers of the pressure (in which we have omitted the subscript 0 from the radius *a* for clarity)

$${\left(u/a\right)}^{2}-(A_{3}/a^{2})p^{3}=(A_{0}/a^{2})+(A_{1}/a^{2})p+(A_{2}/a^{2})p^{2}+(A_{-1}/a^{2})p^{-1},$$ (26)

from which we obtain *A*_0_/*a*^2^ and, ultimately, *T*.

In Eq. (26), the terms with positive integer powers of *p* are related to the virial coefficients that appear in the equation of state of a dilute gas. Thus, the form of these terms has a rigorous derivation, provided that the acoustic frequency is sufficiently low, as is the case here, and provided the acoustic resonance frequencies are not accidentally in near coincidence with a mechanical resonance of the steel shell of the resonator. (A possible coincidence of the (0,6) acoustic resonance with a mechanical resonance is considered in Sec. 8.4.) In Eq. (26), the term (*A*_−1_/*a*^2^)/*p* relaxes the assumption that the thermal accommodation coefficient was exactly one. This assumption was part of the reduction of the resonance frequency data to speed-of-sound data on isotherms described in Sec. 8.1.

Although the pressure on each isotherm was varied by a factor of 20, the values of (*u*/*a*)^2^ on each isotherm varied by less than 0.3 %. At all the pressures used, the dominant term in Eq. (26), (*A*_0_/*a*^2^), is far larger than the other terms. The smallest term, (*A*_3_*p^3^*/*a*^2^), is always less than 3.1 × 10^−6^ of the dominant term (*A*_0_/*a*^2^). Instead of trying to fit such a small term, we fixed the values of *A*_3_ on the left hand side of Eq. (26) to results from Boyes’ speed-of-sound measurements [33]. Boyes’ measurements extended up to 10 MPa where (*A_3_p*^3^/*A*_0_) becomes as large as 0.02. Boyes’ data spanned the range 250 K to 350 K; thus, extrapolation to lower temperatures was required to determine *A*_3_ on our isotherms at 217 K and 234 K. To do so, we fitted a polynomial function of *T* to Boyes’ data. The resulting values of (*A*_3_/*a*^2^) are listed in the right-hand column of Table 7. We assumed that the uncertainty of the extrapolated *A*_3_ was equal to *A*_3_ itself. If this is true, the fractional effect of *A*_3_ on the values of *A*_0_ and on *T* deduced from Eq. (26) is only a few times 10^−7^.

For each argon isotherm, the parameters for Eq. (26) and the relative standard deviation of the fit are listed in Table 7. The mean of the relative standard deviations of the fits is 1.18 × 10^−6^, which corresponds to approximately 0.3 mK. The relative uncertainties of the parameters *A*_0_/*a*_0_^2^ are carried to Rows 4 and 5 of Table 2, where the uncertainties of (*T*−*T*_90_) are computed.

**Fig. 9:**
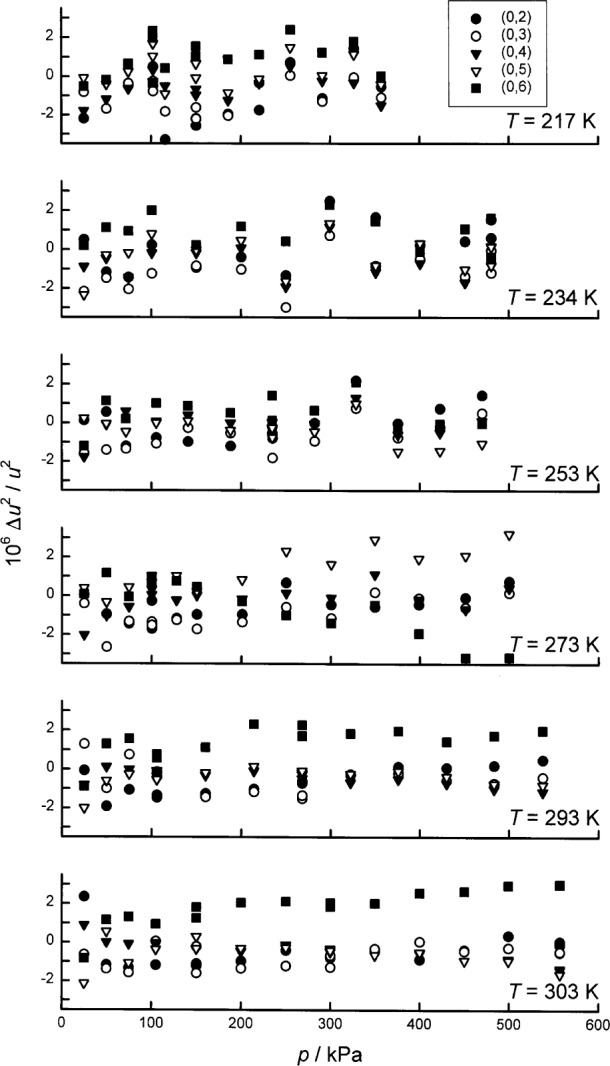
Speed of sound in argon: isotherm analysis. Deviations of the data for the speed of sound in argon from Eq. (26) with the parameters from Table 7 (Δ*u*^2^ ≡ *u*^2^_measured_ − *u*^2^_fitted_).

Figure 9 displays the deviations of the data from Eq. (26) as a function of pressure. On the isotherms at 273 K, 293 K, and 303 K, the deviations are clearly mode-dependent; the data for the (0,6) mode are farthest from the mean of the others, especially at the higher pressures. This will be discussed in Sec. 8.4.

### 8.3 Fitting *u*^2^(*p*,*T*) Surface

The isotherm analysis is one extreme, insofar as it uses the fewest possible assumptions concerning the fitting parameters. Thus, the results do not have uncertainties resulting from choosing only one among several competing models for the data. Here, we consider an alternative that tends towards the opposite extreme: we impose as many physically-based constraints on the parameters as possible. Remarkably, the imposition of these constraints changes (*T*−*T*_90_) on the average by 0.6 mK, and 0.8 mK in the worst case. Furthermore, these changes are within the combined uncertainties of the two analyses (see Table 1). We expect that imposing more constraints than in Sec. 8.2 but fewer constraints than imposed here will lead to results between those resulting from these extremes.

The most significant constraint when fitting *u*^2^(*p*,*T*) was applied to *A*_1_(*T*). We noted that *A*_1_(*T*) ≡ *γ*^0^*β*_a_(*T*)/*M* where *β*_a_(*T*) is the second acoustic virial coefficient, *γ*_0_ = *C_p_*^0^/*C_v_*^0^ = 5/3 is the ideal-gas heat-capacity ratio for argon, and *M* is the molar mass of the argon. One may calculate *β*_a_(*T*) from the argon-argon interatomic pair potential. The semi-empirical HFD-B2 potential function of Ref. [24] has been refined to simultaneously represent information from theory, molecular beam scattering, spectroscopy of argon dimers, measurements of density virial coefficients, and measurements of transport properties. However, it does not make use of speed-of-sound data. Ewing et al. measured *β*_a_(*T*) for argon with an acoustic resonator [34]. Remarkably, their values of *β*_a_(*T*) differed from those computed from the HFD-B2 potential function by a very small, linear function of the temperature in the range from 100 K to 304 K. We used Ewing et al.’s modified HFD-B2 potential function to compute the values *A*_1_(*T*) that served as a fixed base line when fitting the present data (these values appear in Table 8). When fitting our data, we included the term

$$\Delta A_{1}\left(T\right)/{a_{0}}^{2}\equiv b_{0}+b_{1}\left(T-T_{w}\right).$$ (27)

Thus, we constrained the values of *A*_1_(*T*) on six isotherms to be fitted by two parameters, *b*_0_ and *b*_1_, instead of six parameters.

The second constraint when fitting *u*^2^(*p*,*T*) was applied to *A*_2_(*T*). We used the expression

$$A_{2}\left(T\right)/{a_{0}}^{2}\equiv c_{0}+c_{1}/T+c_{2}/T^{2},$$ (28)

thereby constraining the values of *A*_2_(*T*) on six isotherms to be fitted by three parameters: *c*_0_, *c*_1_, and *c*_2_. As mentioned in Sec. 1, the rationale for this constraint begins with the observation that the present isotherms are well above the critical temperature *T*_c_ of argon (1.4 ≤ *T*/*T*_c_ ≤ 2.0) where the viral coefficients of argon are only weakly temperature dependent. Furthermore, the present data are well below the critical density (*ρ*/*ρ*_c_ ≤ 0.02), where the virial expansion converges rapidly. The ratio of terms (*A*_2_*p*^2^/*a*^2^)/(*A*_0_/*a*^2^) attains a maximum value of only 1.5 × 10^−4^; thus, only a moderately precise representation of *A*_2_(*T*) is required.

The final constraint for fitting the *u*^2^(*p*,*T*) surface was to assume that the thermal accommodation coefficient *h* is exactly 1, which is its temperature-independent maximum value. (If we had simply assumed that *h* was independent of temperature, its optimum value would have been 0.90 and the resulting values of *T*/*T*_w_ would have been negligibly different from the present surface fit.)

**Fig. 10:**
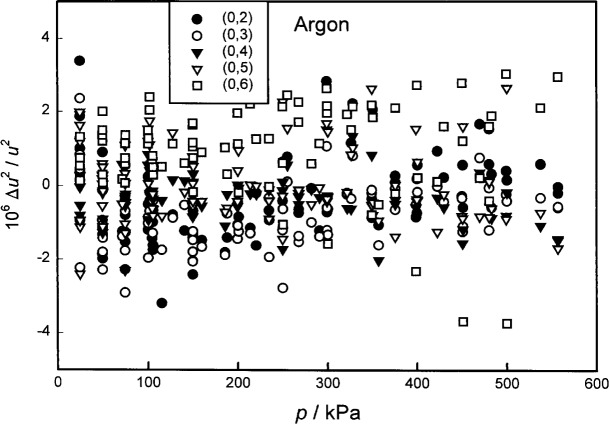
Speed-of-sound in argon: surface analysis. Deviations (Δ*u*^2^ ≡ *u*^2^_measured_ − *u*^2^_fitted_) of the data from Eqs. (26), (27), and (28) with the parameters from Table 8. The results for the different radial modes are denoted (0,2) etc.

The parameters resulting from fitting the *u*^2^(*p*,*T*) surface to the argon data are listed in Table 8. The fractional uncertainties of the parameters *A*_0_/*a*_0_^2^ are carried to Rows 6 and 7 of Table 2, where the uncertainties of (*T*−*T*_90_) are computed. The deviations of the data from the fit are shown in Fig. 10. The deviations are comparable to those obtained from fitting the data on each isotherm separately (See Fig. 9.). The relative standard deviation of the fit was 1.12 × 10^−6^, which is close to 1.18 × 10^−6^, the mean of the relative standard deviations from the isotherm fits. Thus, by statistical criteria, the 11-parameter surface fit to the *u*^2^(*p*,*T*) data is just as valid as the isotherm fits that require 24 parameters.

### 8.4 Alternative Fits of Argon *u*^2^(*p*,*T*) Data

As evident in Fig. 9 for the isotherms at 273 K, 293 K, and 303 K, the results from the (0,6) acoustic mode differ from the average results from the other modes. A similar phenomenon was observed during the previous redetermination of *T*_g_ with this resonator [7]. Then, the (0,6) mode was perturbed more strongly at 10.0 kHz at *T*_g_ than at 9.5 kHz at *T*_w_ and the perturbation was attributed to the near coincidence of the (0,6) frequency with a non-radial shell resonance that had been predicted to occur at 10.15 kHz. Perhaps the intervening modifications of the apparatus, such as attaching the copper shield to the resonator, reduced the frequency of the non-radial shell mode. If this had happened, the near coincidence of the shell mode and the (0,6) mode might occur at the lower temperatures where the frequency of the (0,6) mode was also lower.

In Ref. [7] an additional parameter, *A*_1_^*^, applicable to the (0,6) mode only, was included in the fit to account for the coincidence of that mode with the shell resonance. In this work, by contrast, we elected to repeat both the isotherm and the surface analyses of the acoustic data excluding the (0,6) data entirely. Table 9 compares results from these analyses with the earlier ones. Upon excluding the (0,6) mode, the values of (*T*−*T*_90_) are reduced only about 0.1 mK and the standard deviations of the fits are reduced by approximately 25 %.

For evaluating this result and for future work, it is useful to compare the effect of the compliance of the gas-constant resonator’s shell to the coefficient *A*_1_. This was done in Eq. (3.1) of Ref. [4] for the radially-symmetric (“breathing”) mode of the shell. A radial acoustic mode at frequency *f*_a_ is shifted by Δ*f*_shell_, where

$$\frac{\Delta f_{\text{shell}}}{f_{a}}=\frac{-\gamma_{0}\chi_{s,i}p/3}{1-{\left(f_{a}/13.58\mathrm{kHz}\right)}^{2}},$$ (29)

and where the compliance for internal pressure is *ᵡ*_s,i_ = (3.03 ± 0.03) × 10^−11^ Pa^−1^ at *T*_w_. Far from 13.58 kHz, Δ*f*_shell_ is a linear function of pressure that contributes 17 × 10^−6^ to Δ*u*^2^/*u*^2^ near 500 kPa. If Δ*f*_shell_ were not accounted for, its effects would be obvious on deviation plots, such as Fig. (9). Upon comparing Δ*f*_shell_ to *A*_1_ at, for example, *T*_w_ we find (*ᵞ*_0_*ᵡ*_s,i_/3)/*A*_1_ ≈ 0.007. Thus, far from the resonance, the effect of radial compliance is twice the uncertainty of *A*_1_ as deduced from the isotherm fits and close to a resonance the effect will be much larger. Similar considerations apply to the bending modes of the shell that fall below 13.58 kHz. In cases where a shell resonance is suspected, the resonance could be unambiguously revealed by the acquisition of data on closely spaced isotherms. In the vicinity of the resonance, *A*_1_(*T*) will have an anomalous temperature dependence for the acoustic mode closest to the shell mode. When *f*_a_ is very close to a shell resonance, expressions similar to Eq. (29) must be superceded by more complex ones that account for damping of the shell’s motion and for the “avoided crossing” of the gas’ resonance and the shell’s resonance.

We also considered a surface fit such that Δ*A*_1_(*T*) had the quadratic representation

$$\Delta A_{1}\left(T\right)/a_{0}^{2}\equiv b_{0}+b_{1}\left(T-T_{w}\right)+b_{2}{\left(T-T_{w}\right)}^{2}.$$ (30)

The results fell approximately half way between the surface fit and the isotherm fits with a negligible reduction in the standard deviation of the fit (see Table 9).

The fits that we considered here and the analysis of all the previous results from this resonator weighted the acoustic data with the pressure dependence given by (*σ*_1_^2^ + *σ*_2_^2^)^−1^, where *σ*_1_ and *σ*_2_ are given by Eq. (25) and the text associated with that equation. However, the signal-to-noise ratio of the present data does not decrease with pressure according to Eq. (25) and previous experience with this apparatus [see Fig. (10)]. Presumably, the improvements mentioned in Sec. 4 account for the change. In future work, we will investigate the effects of weighting the acoustic data independently of the pressure and we will implement the improvements enumerated in Sec. 1.1. We hope that these changes in the apparatus, the procedures, and the weighting of the data will determine the parameter (*A*_0_/*a*^2^) in Eq. (26) more precisely. Perhaps these changes will reduce the differences between the surface fits and the isotherm fits in Table 9. If so, the overall uncertainties in (*T*−*T*_90_) might be reduced.

### 8.5 Acoustic Data for Xenon

#### 8.5.1 The Virtual Leak

The most important problem encountered when analyzing the acoustic data for xenon was the progressive change in the speed of sound in the gas while the gas was in the resonator. Because there were no known leaks in the gas handling system, we attributed the contamination to a virtual leak, that is, a volume sealed from the laboratory and connected to the resonator by a path of low pumping speed. If such a volume were exposed to a contaminating gas at high pressure, it would fill rapidly via Poiseuille flow. Subsequently, such a volume would be a lingering source of contamination because it would evacuate comparatively slowly via molecular flow. A precursor to this problem was detected during the redetermination of *R*. We quote from Ref. [4]: “When the resonator was filled with helium, we measured a slow decrease in the resonance frequencies. In a typical case the fractional decrease was 9.3 × 10^−6^/(100 h) with 438 kPa of He-M in the resonator… We speculate that slow desorption of impurities is responsible for these effects. Possible sources of water etc. are the “Viton” O-rings which seal the microphone ports and the fill port to the resonator.” To this list of possible sources, we add the wax used to seal the two hemispheres together.

During the present measurements, when the resonator was filled with xenon at *T*_g_, the resonance frequencies slowly *increased*. In a typical case, the fractional increase was 2 × 10^−6^/(60 h) at *T*_g_ with 150 kPa of Xe in the resonator. Presumably, the xenon was progressively contaminated with an impurity with a higher speed of sound. Again, we suspected that the impurity had been dissolved in the O-rings. Perhaps, for example, argon had diffused into the O-rings comparatively rapidly while the resonator was filled with argon at high pressure. Later, when the argon in the resonator was replaced by xenon, small amounts of argon slowly desorbed from the O-rings into the resonator, driven only by the concentration gradient remaining within the O-rings. This speculation is consistent with measurements conducted with argon immediately following the xenon measurements. Then, a progressive fractional *decrease* in the resonance frequencies of 4 × 10^−6^/(40 h) was measured at *T*_g_ with 150 kPa of argon in the resonator. The change in sign is consistent with the argon sample being progressively contaminated by desorbing xenon and the larger rate is reasonable considering that (1/*u*^2^)(d*u*^2^/d*x*) is 3.3 times larger for dilute xenon in argon than for dilute argon in xenon. In contrast, progressive contamination was not detected during similar measurements at *T*_w_ with either argon or xenon in the resonator. We do not know whether the capacity of the O-rings is much lower at *T*_w_ and/or the rate of desorption is so slow (or so rapid) that we did not observe it.

We emphasize that when the resonator was filled with argon *prior* to the xenon measurements, we never observed a secular change in the frequencies at any of the temperatures under study.

#### 8.5.2 Correction of the Xenon Data for Contamination

In order to cope with the progressive contamination of the xenon at *T*_g_, we decided to measure the resonance frequencies as a function of time *t* at each pressure. The values of *f*_a_(*t*) were extrapolated backward to the time when the resonator was first filled and when, presumably, the contamination began. To minimize the uncertainty resulting from the extrapolation, *f*_a_(*t*) was measured at *T*_g_ for three separate fillings of the resonator. This greatly reduced the residence time of each sample in the resonator and hence its progressive contamination. Furthermore, the resonator was evacuated and “baked” between successive fillings.

At each temperature and pressure, the values of the derived speeds of sound from each mode were averaged. The averages were corrected for progressive contamination assuming that, for each sample, the contamination effect was inversely proportional to the pressure and directly proportional to the sample’s residence time at each pressure. For each sample, successive corrections for the different pressures were assumed to be additive and the time required to reduce the pressure was neglected in comparison with the residence time at any particular pressure. Thus, the correction Δ*u* for the *i*-th pressure is

$$\Delta u_{i}=\sum_{j=1}^{i}kt_{j}/p_{j}$$ (31)

in which *t_j_* is the time spent at pressure *p_j_*. We did *not* assume that the outgassing-rate-constant *k* was the same for each sample. Instead, we assumed that it was independent of pressure and time for a given sample and we determined it by monitoring the drift of the resonance frequencies at constant temperature and at a low pressure for at least 12 h. The values of *k* for each of the three samples are listed in the right column of Table 10. Table 10 also contains the averaged values of *u*(*p*,*T*_g_,*t*) and the values extrapolated to *t* = 0.

**Fig. 11:**
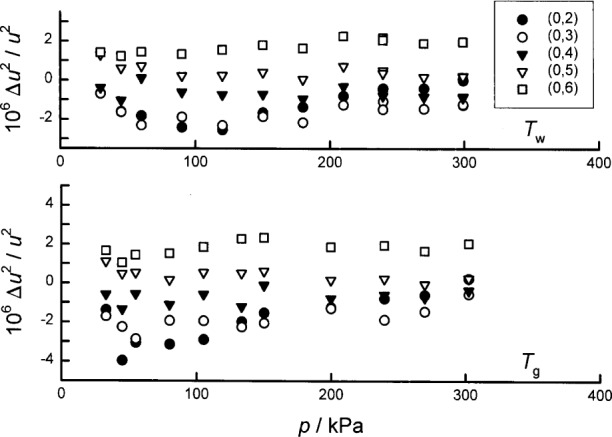
Speed-of-sound in xenon. Deviations (Δ*u*^2^ ≡ *u*^2^_measured_ − *u*^2^_fitted_) of the data from Eq. (26) with *A*_−1_ ≡ 0 and with the other parameters from Table 11.

The extrapolation procedure was successful. The deviations of the xenon data at *T*_g_ from fitting functions do not show discontinuities where the samples were changed (see below and Fig. 11). The maximum fractional correction to *u*^2^ for the virtual leak was 7.6 × 10^−6^ with an uncertainty of approximately 0.24 × 10^−6^. If the correction had been neglected, the derived value of *T*_g_/*T*_w_ would have been erroneous because of an incorrect determination of the curvature of the isotherm.

#### 8.5.3 Fitting to Xenon Acoustic Data

The xenon data for the isotherms at *T*_w_ and *T*_g_ were corrected for all of the phenomena that were discussed in Sec. 8.1 in connection with fitting the argon data for the six isotherms. Then the xenon data were averaged and corrected for the virtual leak. The resulting values of *u*/*a*_0_ averaged over five modes are listed in Table A3 in the Appendix.

Equation (26) was independently fitted to the corrected data for each isotherm, as discussed in Sec. 8.2, except that the characteristic pressure in the weighting function [see Eq. (25)] was replaced by 5.5 × 10^4^ Pa and the value of *A*_3_ was fixed at zero. (The maximum value of (*A*_3_/*A*_0_)*p*^3^ is only 9 × 10^−6^ in the present work. This was determined by estimating *A*_3_ for xenon from a dimensionless plot of (*A*_3_/*A*_0_)(*Tp*_c_/*T*_c_)^3^ as a function of *T*/*T*_c_ that we made with acoustically-determined values of *A*_3_ for argon [33] and xenon [35].)

Because there were only two xenon isotherms, we considered only one constraint; namely, fixing the thermal accommodation coefficient at unity or, equivalently, requiring *A*_−1_ to be identically 0. Upon allowing *A*_−1_ ≠ 0, the standard deviation of the fits increased and the best values of *A*_−1_ did not change from zero within their uncertainties. Thus, both the xenon data and the argon data are consistent with *A*_−1_ identically equal to 0.

The parameters and the standard deviations resulting from the fits are listed in Table 11. Figure 11 shows the deviations of the xenon data from Eq. (26) for the constrained fits at *T*_w_ and *T*_g_. For the constrained fit, the uncertainties in *A*_0_/*a*^2^ contribute 1.3 × 10^−6^ to the uncertainty in *T*_g_/*T*_w_; for the unconstrained fit, the contribution is 4.6 × 10^−6^. When the non-acoustic sources of uncertainty (Table 2) are added in quadrature, the constrained xenon fit leads to the result (*T*_g_−*T*_90_) = 4.38 ± 0.48 mK and the unconstrained fit leads to (*T*_g_−*T*_90_) = 4.38 ± 1.42 mK.

Most of the deviations in Fig. 11 are independent of pressure and they do not show discontinuities at the pressures where the resonator was refilled. The deviations do depend upon the mode index in a similar manner on both isotherms. This led us to consider small adjustments to the eigenvalues that could be rationalized by our imperfect knowledge of the geometry of the resonator. Such adjustments reduced the standard deviation of the xenon fits by a large factor. However, the same adjustments had essentially no effect on the argon surface fit. We concluded that the xenon deviations are a measure of our ignorance of the resonator’s behavior. It may be useful to reconsider this matter in the future, when argon data become available on closely spaced isotherms. Such data may separate the effects of imperfect knowledge of the eigenvalues from imperfect knowledge of the shell’s dynamic compliance.

## 9. Comparisons with Previous Acoustic Measurements

### 9.1 Comparison at *T*_w_

**Fig. 12:**
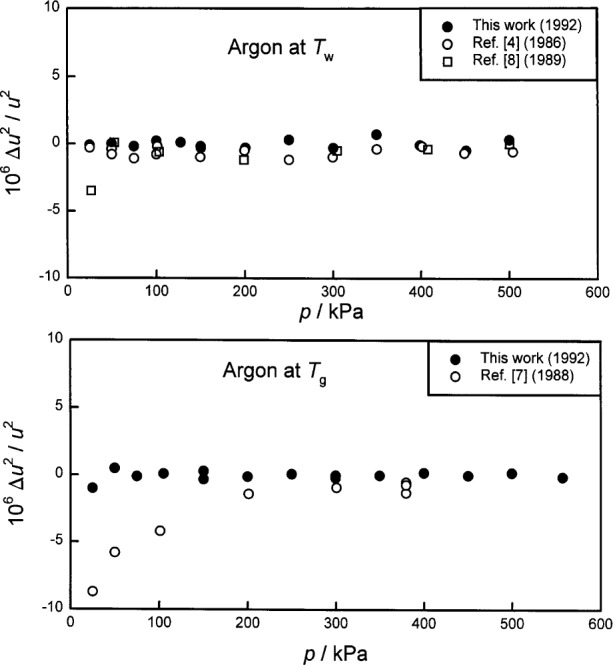
Comparison of present results with previous results obtained with the same apparatus (Δ*u*^2^ ≡ *u*^2^_measured_ − *u*^2^_fitted_). Top: *u*^2^_fitted_ for this plot came from the redetermination of *R* [4]. Bottom: *u*^2^_fitted_ is Eq. (26) with the parameters for *T*_g_ from Table 8. The “Ref. [7] 1988” data were used to remeasure *T*_g_ and are now suspect.

In the upper panel of Fig. 12, the present results at *T*_w_ are compared with previous measurements made with the same gas in the same apparatus. The baseline is the fit to the isotherm used to redetermine *R* and reported in Table 11 of Ref. [4]. Obviously, the differences between the present results and the measurements conducted in 1986 are very small; the average difference is on the order of, fractionally, 1 × 10^−6^ over the entire pressure range. It is possible that some of this difference could be explained by recalling that, following the 1986 measurements, a transducer was relocated resulting in a possible decrease in the volume of the resonator by as much as 0.88 × 10^−6^, fractionally (see Sec. 9.6 of Ref. [4]). This corresponds to a 0.59 × 10^−6^ fractional increase in *u*^2^. If so, the discrepancy is within the experimental uncertainty.

The upper panel of Fig. 12 also displays the unpublished results of 1989 [8]. They are close to both the present results and those of 1986. This figure provides convincing evidence that the volume of the gas-constant resonator has been very stable for many years.

### 9.2 Comparison at *T*_g_

The lower panel of Fig. 12 compares the present results for *u*^2^ at *T*_g_ with the earlier ones obtained with the same apparatus. For this panel, the baseline is Eq. (26) with the present parameters from Table 8. The present measurements nearly agree with the unpublished results of 1989. The maximum fractional difference is only 2 × 10^−6^ at the highest pressure. This difference decreases approximately linearly with pressure, and the zero-pressure values of *u*^2^ differ by less than 1 × 10^−6^ *u*^2^, which is equivalent to only 0.3 mK in (*T*−*T*_90_). This is remarkably good agreement.

In contrast, the data labeled “Ref [7] 1988” from the previous redetermination of *T*_g_ are seen to be in poor agreement with the present results. At the highest pressure the results agree to better than 2 × 10^−6^, fractionally. However, as the pressure decreases, the difference grows non-linearly, reaching approximately 9 × 10^−6^, fractionally, at 25 kPa. We now believe that this was a result of progressive contamination of the argon sample, similar to the contamination of the present xenon samples and the earlier helium samples that we described in Sec. 8.4.1.

One question remains. Common impurities *increase* the speed of sound in argon; however, the departure of the earlier data from the present data is consistent with a progressive *decrease* of the speed of sound. The question is: what impurity could have caused this decrease?

We considered the possibility that a droplet of mercury remained in the resonator from the weighings associated with the redetermination of *R*. The saturated vapor pressure of mercury is 0.37 Pa at *T*_g_ and (1/*u*^2^)(d*u*^2^/d*x*) = − 4 for a mercury impurity in argon. Thus, mercury at its saturated vapor pressure would fractionally decrease the speed of sound in argon at 100 kPa by 15 × 10^−6^, much more than was observed.

We note from Sec. 9.1 of Ref. [7] that helium was in the resonator prior to the argon measurements in question and that the helium measurements were followed by baking out the resonator under vacuum for 4 d at 60 °C and two flushes with argon. This probably was the first time that the gas constant resonator had been maintained at such a high temperature since it was assembled. Perhaps some moderately heavy hydrocarbons diffused out of the wax that sealed the two hemispheres together and dissolved into the O-rings. The experience with xenon indicates that flushing did not remove the source of the contamination. Perhaps the same phenomena occurred during the “Ref [7] 1988” argon measurements.

## 10. Checks for Systematic Errors

In this section we consider tests for systematic errors. In the first test, the isotherm parameters determined in this work are compared with previously published values. In the second, the measured half-widths are compared with those calculated from the theory. In the third test, the molar mass of xenon calculated from the present results is compared with the value determined from IUPAC tables of the isotopic composition of xenon. In all cases we find satisfactory agreement.

### 10.1 Discussion of the Isotherm Parameters

#### 10.1.1 The Regression Coefficient *A*_1_(*T*)

**Fig. 13:**
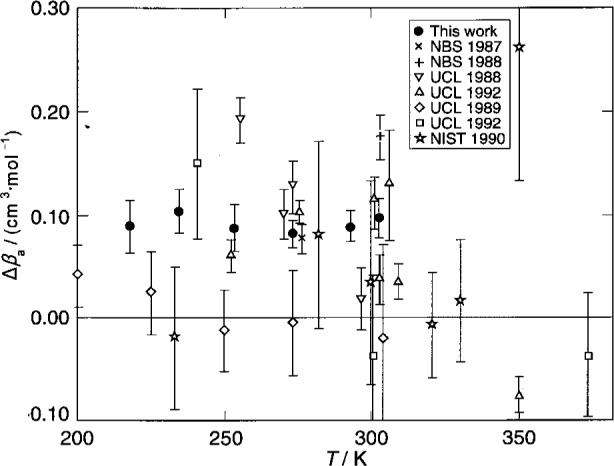
Deviation of experimental values of *βb*_a_(*T*) from the values calculated from the potential of Ref. [34]. Key: ●, this work; ×, Ref. [4]; +, Ref. [7]; ∇,, Ref. [37]; Δ, Ref. [33]; ◊, Ref. [35]; □, Ref. [35]; ☆, Ref. [38].

For comparisons, we consider the more familiar second acoustic virial coefficient *β*_a_(*T*) = *RTA*_1_(*T*)/*A*_0_(*T*). In Fig. 13, we compare the values of Δ*β*_a_ = [*β*_a_(*T*)_measured_ − *β*_a_(*T*)_calculated_] from our independent isotherm analysis (Table 8) and from other measurements. The baseline of Fig. 13 is the values of *β*_a_(*T*) that were calculated from the HFD-B2 intermolecular potential of Ewing and Trusler [34]. This potential was adapted by Ewing et al. [36] to fit their own measurements of *β*_a_(*T*) that are labeled “UCL 1989” on the figure. The uncertainty of the baseline must be on the order of the experimental uncertainty associated with their results (rms uncertainty of 0.081 cm^3^·mol^−1^).

In the present work, the largest contribution to the uncertainty of *β*_a_(*T*) is the standard deviation of *A*_1_(*T*) from the isotherm analysis. The standard deviations in Table 7 propagate into uncertainties of 0.010 cm^3^·mol^−1^ to 0.022 cm^3^·mol^−1^ for *β*_a_(*T*). The present values of *β*_a_(*T*) are subject to two sources of uncertainty arising from systematic effects that were discussed in connection with the redetermination of *R* [4]. First, the uncertainty of the shell’s compliance *ᵡ*_s,i_ was estimated to be 6 % of its value, independent of the temperature. This propagates into an uncertainty of 0.085 % of *A*_1_(*T*_w_) and it is equivalent to an uncertainty of 0.0046 cm^3^·mol^−1^ in *β*_a_(*T*). Second, the uncertainty of the compliance of the transducers propagates into an uncertainty of 0.0025 cm^3^·mol^−1^ in *β*_a_(*T*). We calculated the standard uncertainty of *β*_a_(*T*) from the sum in quadrature of these two terms and the standard deviation of *A*_1_(*T*) from the fit. We also included terms from the uncertainties of *h* and of the thermophysical properties required in the calculations; these contribute less than 0.01 cm^3^·mol^−1^ to the uncertainty of *β*_a_(*T*).

Figure 13 shows that the present results differ from the potential of Ewing and Trusler [34] by approximately 0.09 cm^3^·mol^−1^ across the whole range. This is barely outside the combined uncertainties. The value determined at *T*_w_ differs from the value from [4] by only 0.009 cm^3^·mol^−1^. This level of agreement is truly remarkable. In contrast, this determination of *β*_a_ at *T*_g_ differs from that of Ref. [7] by 0.18 cm^3^·mol^−1^. The results determined from the high pressure measurements of Ewing and Goodwin [37] are shown together with results from Goodwin and Moldover [38], and from Boyes [33]. The agreement is better than 0.2 cm^3^·mol^−1^ over the whole range. This level of agreement is extraordinary considering the pressure ranges under study differ by more than a factor of 10.

#### 10.1.2 The Regression Coefficient *A*_2_(*T*)

**Fig. 14:**
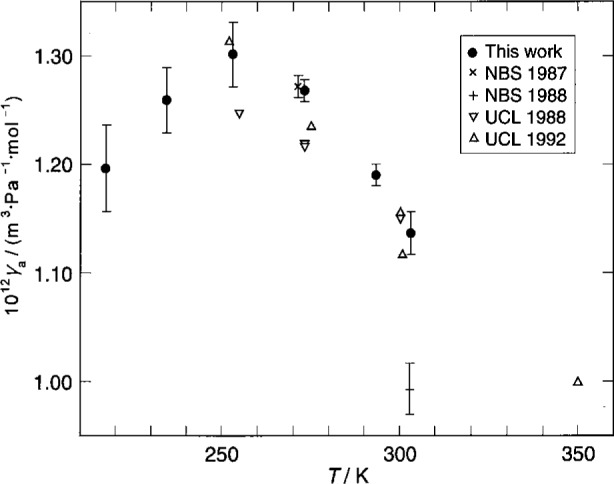
Comparison of values of *γ*_a_(*T*). Key: ●, this work; ×, Ref. [4]; +, Ref. [7]; ∇, Ref. [37]; Δ, Ref. [33].

For comparisons, we converted the present values of *A*_2_(*T*) to the third acoustic virial coefficient *γ*_a_(*T*) = *RTA*_2_(*T*)/*A*_0_(*T*). In Fig. 14, we compare the values of *γ*_a_(*T*) determined in this work with previous values. The value of *γ*_a_(*T*_w_) measured when *R* was redetermined [4] agrees with the present value to within the experimental uncertainty. The results of Ewing and Goodwin [37], and of Boyes [33], which extend to pressures far greater than were studied here, agree with the present results to within twice the combined experimental uncertainties. However, the analysis of the present data incorporated Boyes’ values of *A*_3_; thus, the measurements are not completely independent. The value of *γ*_a_(*T*_g_) from [7] labeled “NBS 1988” is not consistent with the other values of *γ*_a_(*T*). This is another indication that the Moldover-Trusler redetermination of *T*_g_ was in error.

### 10.2 Half-Widths of the Resonances

**Fig. 15:**
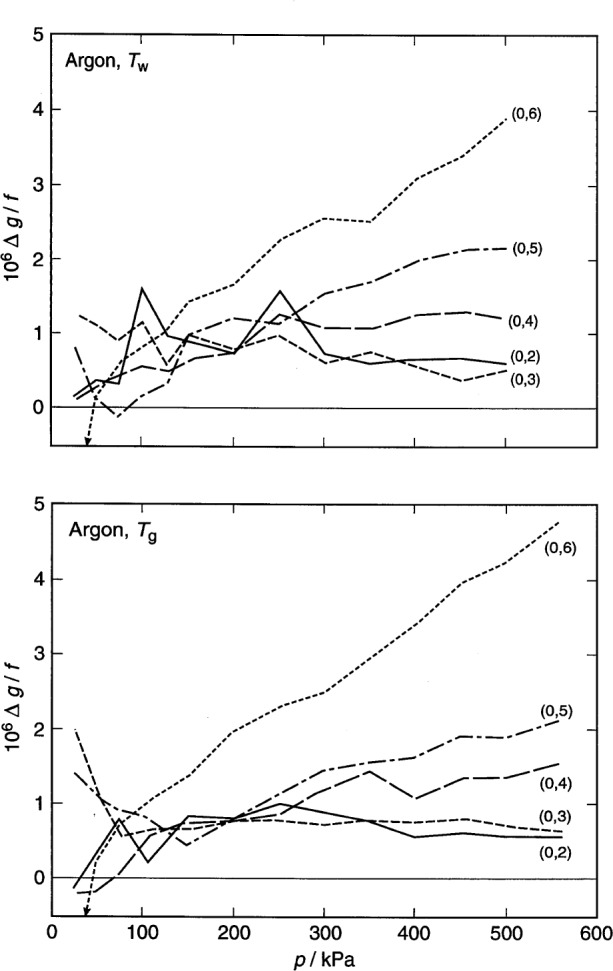
Scaled excess half-widths of the resonances in argon as a function of the pressure (Δ*g* ≡ *g*_measured_ − *g*_calculated_).

At low pressures, the largest correction to the measured resonance frequencies is determined by the acoustic boundary layer and this same boundary layer is also the largest contributor to the half-widths of the acoustic resonances. Thus, the measurements of the half-widths provide an important method for confirming the applicability of the theory of the boundary layer to the present determination of *T*/*T*_w_. We define the excess half-width Δ*g* as the amount by which the measured half-width exceeds the calculated half-width. Figure 15 displays Δ*g* for five radial modes in argon at *T*_w_ and *T*_g_. (The values of Δ*g* are scaled by 10^6^/*f*_a_, where *f*_a_ is the resonance frequency of the mode under study.) For each mode on Fig. 15, an extrapolation to *p* = 0 yields values of Δ*g*/*f* < 10^−6^. This is strong evidence that the boundary-layer correction is applicable to the present data at the lower pressures. The results on Fig. 15 are similar to those observed previously with either argon or helium in this resonator at *T*_w_ and at *T*_g_ and similar to the results obtained in this work on the isotherms below *T*_w_.

**Fig. 16:**
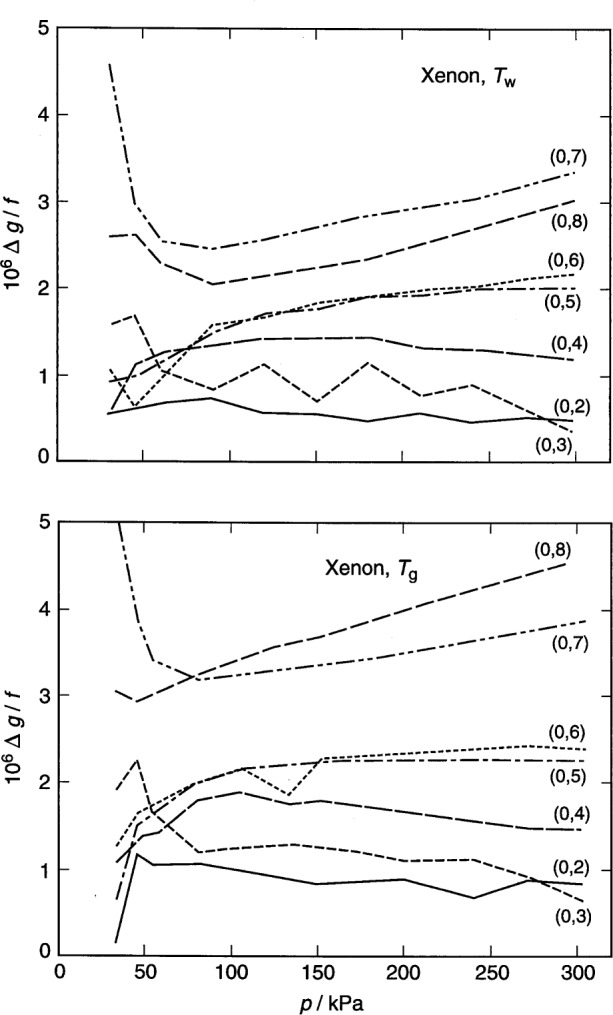
Scaled excess half-widths of the resonances in xenon as a function of the pressure (Δ*g* ≡ *g*_measured_ − *g*_calculated_).

Figure 16 displays Δ*g*/*f* for the measurements with xenon at *T*_w_ and *T*_g_. At low pressures, Δ*g*/*f* of the (0,7) mode increases sharply as a result of partial overlap with the neighboring non-radial (13,2) multiplet (see Fig. 8 of Ref. [4]). The data for the (0,8) mode also indicate a problem upon extrapolation to *p* = 0. For the five lower modes, Δ*g*/*f* < 2 × 10^−6^ at the lower pressures. This bound is small enough to indicate that the boundary-layer correction for the xenon data is not so seriously in error as to vitiate the current redetermination of *T*_g_/*T*_w_ with xenon. However, the pressure dependence of Δ*g*/*f* is more complicated than that for argon and may be a worthy subject for further study, especially under circumstances where progressive contamination is not an issue.

### 10.3 Molar Mass of Xenon

The regression parameter *A*_0_ = *u*_0_^2^ = *γ*^0^*RT*/*M* was determined at both *T*_w_ and *T*_g_ for both argon and xenon. From these values it is possible to determine the ratio of the molar mass of our working xenon sample *M*_wXe_ to the molar mass of our working argon sample *M*_wAr_. We have two estimates of *M*_wXe_/*M*_wAr_ and the difference between them is an indication of uncertainty, probably resulting from the contamination of the xenon. We find *M*_wXe_/*M*_wAr_ = 3.285 957 ± 0.000 004 from the measurements at *T*_w_; we find *M*_wXe_/*M*_wAr_ = 3.285 949 ± 0.000 005 from the measurements at *T*_g_. The fractional difference between these ratios is 2.4 × 10^−6^ which of course is the fractional difference between the values of *T*_g_/*T*_w_ determined with xenon and with argon.

The molar mass of our argon sample was determined previously [4] with a relative uncertainty of only 0.8 × 10^−6^ by comparison with a sample of chemically purified, nearly mono-isotopic ^40^Ar. Using the value of (23.968 684 ± 0.000 019) g/mol for *M*_wAr_/*γ*^0^ we find *M*_wXe_/*γ*^0^ = (78.759 98 ± 0.000 23) g/mol, where we have averaged the two ratios. Our sample of xenon contained krypton with a concentration of 18 × 10^−6^, by mole fraction. Accounting for this leads to a value for the molar mass of Xe of (131.267 49 × 0.000 33) g/mol where we have taken *γ*^0^ = 5/3 exactly. Within combined uncertainties, this value agrees with the value of (131.29 ± 0.03) g/mol recommended by IUPAC. Reference [39] states that “modified isotopic compositions may be found in commercial material … because the material has … inadvertently been subjected to isotopic separation” leading to systematic differences in the estimated molar masses of various different samples. On the scale of the IUPAC uncertainty, our problems associated with contamination of the xenon are minor indeed.

## Figures and Tables

**Table 1:** The difference *T*−*T*_90_

	Isotherm fits linear Δ*A*_1_(*T*)	Surface fit	Recommended
*T*_90_/K	(*T*−*T*_90_)/mK	(*T*−*T*_90_)/mK	(*T*−*T*_90_)/mK
Argon			
302.9166	3.95±0.73	4.61±0.33	4.6±0.6
293.1300	2.60±0.75	3.23±0.31	3.2±0.6
253.1500	−2.84±0.60	−2.29±0.27	−2.3±0.6
234.3156	−3.73±0.60	−2.92±0.19	−2.9±0.6
217.0950	−4.03±0.67	−3.55±0.29	−3.6±0.6

Xenon^a^			
302.9166		4.38±0.66	

a For xenon, the uncertainty is the quadrature sum of 0.40 mK from fitting the acoustic data, 0.31 mK from the non-acoustic items in Table 2, and 0.43 mK from the virtual leak correction.

**Table 2:** Standard uncertainties *u*_s_×10^6^ from various sources in the re-determination of (*T*−*T*_90_)/*T*. The square root of the sum of the squares (RSS) is calculated twice: first, including Rows 4 and 5, but not Rows 6 and 7; and second, including Rows 6 and 7, but not Rows 4 and 5

Source	217 K	234 K	253 K	293 K	303 K
Microwave values for [*a*(*T*)/*a*(*T_w_*)]^2^					
1. Discrepancies among triplets	0.32	0.27	0.25	0.27	0.33
2. δ_m_(*T*) calc. from resistivity (0.04×*ρ*)	0.39	0.25	0.12	0.12	0.18
3. δ_m_(*T*) calc.−δ_m_(*T*) meas.	0.24	0.24	0.24	0.24	0.24

Acoustic (isotherm fits)					
4. Uncertainty of *A*_0_(*T*_w_)/*a*^2^	1.67	1.67	1.67	1.67	1.67
5. Uncertainty of *A*_0_(*T*)/*a*^2^	2.31	1.85	1.35	1.67	1.43

Acoustic (surface fit)					
6. Uncertainty of *A*_0_(*T*_w_)/*a*^2^	0.25	0.25	0.25	0.25	0.25
7. Uncertainty of *A*_0_(*T*)/*a*^2^	0.52	0.38	0.36	0.31	0.38

Thermometry					
8. SPRT & bridge repeat. @ *T*_w_ (10 μΩ)	0.46	0.43	0.40	0.34	0.33
9. Difference between calibrations	0.22	0.15	0.10	0.54	0.78
10. Temperature gradient	0.46	0.43	0.40	0.34	0.33
11. Non-uniqueness of ITS-90	0.9	0.0	0.8	0.6	0.0

Additional sources					
12. Thermal conductivity (0.3 %)	0.20	0.20	0.20	0.20	0.20
13. Uncertainty of pressure zero	0.21	0.12	0.05	0.03	0.07

14. Isotherm fits: RSS	3.13	2.62	2.40	2.58	2.43

15. Surface fit: RSS	1.42	0.92	1.16	1.11	1.13

16. Isotherm fits: RSS×(*T*/mK)	0.68	0.61	0.61	0.76	0.74

17. Surface fit: RSS×(*T*/mK)	0.31	0.22	0.29	0.33	0.34

**Table 3:** Dimensions of hemispheres (in mm) at 20.1 8C

	Lower hemisphere			Upper hemisphere		
Measurement series	*R*_c_	*R*_s_	*h*_s_	*R*_c_	*R*_s_	*h*_s_
R1985	107.960	88.935		107.958	88.918	
P1997	107.967	88.9133	0.0585	107.968	88.8901	0.0554
F1997		88.915	0.056		88.896	0.041

**Table 4:** Volumetric expansion of the cavity between *T*_w_ and *T*_g_

Mode	10^6^[*V*(*T*_g_)/*V*(*T*_w_)−1]	Reference
TM11	1418.7^a^	[6]
	1418.7	This work
TM12	1418.9^a^	[6]
	1419.3	This work
TM13	1419.2	This work
Mercury dilatometry	1416.6±1.5	[6]

a Recalculated using *g*_m_ from Eqs. (13), (14), and (15).

**Table 5:** Record of calibration of thermometers

Date	Thermometer	*R*(*T*_t_,*i* → 0)/Ω
t = Water		
07/04/92	LN1888002	25.541502
28/05/92	LN1888002	25.541497
27/08/92	LN1888002	25.541518
23/10/92	LN1888002	25.541527
07/04/92	LN303	25.475522
28/05/92	LN303	25.475521
27/08/92	LN303	25.475542
23/10/92	LN303	25.475532
28/05/92	RS18A-5	25.206136
27/08/92	RS18A-5	25.206148
23/10/92	RS18A-5	25.206148

t = Gallium		
28/05/92	LN1888002	28.558768
27/08/92	LN1888002	28.558760
28/05/92	LN303	28.484839
27/08/92	LN303	28.484831
28/05/92	RS18A-5	28.182858
27/08/92	RS18A-5	28.182863

t = Mercury		
28/05/92	LN1888002	21.560995
27/08/92	LN1888002	21.561004
28/05/92	LN303	21.505550
27/08/92	LN303	21.505572
28/05/92	RS18A-5	21.279150
27/08/92	RS18A-5	21.279155

t = Argon		
23/10/92	LN1888002	5.515220
23/10/92	LN303	5.502255
23/10/92	RS18A-5	5.449206

**Table 6:** Summary of thermometer characteristics

Thermometer	LN1888002	LN303	RS18A-5
*T*/K ≤ *T*_w_ (May 1992)			
10^5^*a*	−8.545941	−14.787059	−40.359244
10^6^*b*	2.636814	3.918078	6.610082

*T*_w_ < *T*/K ≤ *T*_g_ (May 1992)			
10^4^*a*	−1.245599	−1.774805	−4.376422

*T*/K ≤ *T*_w_ (August 1992)			
10^5^*a*	−8.299809	−14.871504	−40.211829
10^6^*b*	4.242863	3.367348	7.572838

*T*_w_ < *T*/K ≤ *T*_g_ (August 1992)			

10^4^*a*	−1.350379	−1.879442	−4.406030

**Table 7:** Parameters from analysis of the acoustic isotherms of argon

*T* (K)	*A*_0_/*a*_0_^2^ (s^−2^)	10^6^ *A*_1_/*a*_0_^2^ (s^−2^·Pa^−1^)	10^9^ *A*_2_/*a*_0_^2^ (s^−2^·Pa^−2^)	10^−4^ *A*_−1_/*a*_0_^2^ (s^−2^·Pa)	10^6^ *σ* (*u*^2^)/*u*_0_^2^	10^18^ *A*_3_/*a*_0_^2^ (s^−2^·Pa^−3^)
302.9166	13 282 850 ± 19	66 121 ± 95	6.01 ± 0.12	76 ± 94	1.28	60
293.1300	12 857 689 ± 16	54 820 ± 83	6.25 ± 0.11	74 ± 76	1.03	87
273.1600	11 989 200 ± 20	28 513 ± 109	6.73 ± 0.16	18 ± 94	1.32	147
253.1500	11 117 708 ± 15	−2 868 ± 83	6.87 ± 0.16	84 ± 65	0.88	216
234.3156	10 296 361 ± 19	−38 619 ± 98	6.64 ± 0.14	58 ± 85	1.19	293
217.0950	9 544 464 ± 22	−78 398 ± 150	6.27 ± 0.29	59 ± 89	1.21	374

**Table 8:** Parameters from the *u*^2^(*p*,*T*) surface analysis for argon. The thermal accommodation coefficient *h* was constrained to be exactly 1. *A*_1_(*T*) differs from *A*_1_(*T*) predicted by the HFD-B2 interatomic potential by a fitted linear function [Eq. (27)] of *T*. Also, *A*_2_(*T*) is fitted by a quadratic function [Eq. (28)] of 1/*T*.

*T* (K)	*A*_0_/*a*_0_^2^ (s^−2^)	10^6^ *A*_1_/*a*_0_^2^ (s^−2^·Pa^−1^)	10^18^ *A*_3_/*a*_0_^2^ (s^−2^·Pa^−3^)
302.9166	13 282 869 ± 5	64 879	60
293.1300	12 857 707 ± 4	53 520	87
273.1600	11 989 191 ± 3	27 191	147
253.1500	11 117 724 ± 4	−4 437	216
234.3156	10 296 389 ± 4	−40 442	293
217.0950	9 544 478 ± 5	−80 295	374
	10^4^*b*_0_ = (13.67 ± 0.23) s^−2^·Pa^−1^		
	10^6^*b*_1_ = (−7.31 ± 0.77) s^−2^·Pa^−1^·K^−1^		
	10^9^*c*_0_ = (−15.31 ± 0.90) s^−2^·Pa^−2^		
	10^6^*c*_1_ = (10.91 ± 0.46) s^−2^·K·Pa^−2^		
	10^3^*c*_2_ = (−1.339 ± 0.061) s^−2^·K^2^·Pa^−2^		

**Table 9:** The difference *T*−*T*_90_

	Surface fits			Isotherm fits	

	Sec. 8.3	(0,6) omitted	quadratic Δ*A*_1_(*T*)	Sec. 8.2	(0,6) omitted
10^6^*σ* (*u*^2^)/*u*_0_^2^	1.12	0.84	1.11	1.18	0.85

*T*_90_/K			*T*−*T*_90_		

302.9166	4.61	4.48	4.25	3.95	3.82
293.1300	3.23	3.10	3.06	2.60	2.56
253.1500	−2.29	−2.43	−2.34	−2.84	−2.89
234.3156	−2.92	−3.06	−3.12	−3.73	−3.72
217.0950	−3.55	−3.65	−3.92	−4.03	−4.23

**Table 10:** Virtual leak correction for xenon at 302.91 K

Run no.	*p* (kPa)	*t* (h)	〈*u*/*a*〉*_t_* (s^−1^)	10^6^ δ*u*	〈*u*/*a*〉*_t_*_→0_ (s^−1^)	10^6^ *σ*	10^6^ *k* (kPa·h^−1^)
1	33.095	5.	2009.2915		2009.2912	0.86	
				1.46±0.64			3.32
	33.065	19.5	2009.2952				
2	302.569	16.	1998.9358		1998.9355	0.02	
	269.851	19.	2000.1993		2000.1990	0.03	
	239.836	22.	2001.3573		2001.3569	0.04	
	199.948	25.	2002.8936		2002.8931	0.05	
	150.100	28.5	2004.8097		2004.8092	0.06	
				0.95±0.22			2.39
	150.038	88.	2004.8141				
3	133.515	13.5	2005.4467		2005.4456	0.13	
	105.227	17.	2006.5318		2006.5302	0.17	
	79.949	20.5	2007.5001		2007.4980	0.23	
	54.958	23.8	2008.4570		2008.4542	0.29	
				1.36±0.30			5.75
	54.941	36.5	2008.4600				
	44.964	40.8	2008.8429		2008.8364	0.62	

**Table 11:** Parameters from analysis of the acoustic isotherms of xenon

*T* (K)	*A*_0_/*a*_0_^2^ (s^−2^)	10^6^ *A*_1_/*a*_0_^2^ (s^−2^·Pa^−1^)	10^9^ *A*_2_/*a*_0_^2^ (s^−2^·Pa^−2^)	10^−4^ *A*_−1_/*a*_0_^2^ (s^−2^·Pa)	10^6^ *σ*(*u*^2^)/*u*_0_^2^
273.1600	3 648 625.6±3.3	−204 000±39	−8.91±0.10	0	1.28
302.9166	4 042 322.7±3.7	−153 363±46	−1.93±0.13	0	1.40
273.1600	3 648 617.1±11.5	−203 940±86	−9.03±0.18	31±39	1.28
302.9166	4 042 319.8±14.1	−153 342±111	−1.98±0.24	10±47	1.51

## Footnotes

3 Certain commercial equipment, instruments, or materials are identified in this paper to foster understanding. Such identification does not imply recommendation or endorsement by the National Institute of Standards and Technology, nor does it imply that the materials or equipment identified are necessarily the best available for the purpose.

## 11. Appendix A. Tabulated Data

**Table A1:** Frequencies of microwave resonances

TM*lm*	*f*_d_/MHZ	*f*_s_/MHz	〈*f*_m_〉/MHz	*g*_m_(expt) (MHz)	*g*_m_ (calc) (MHz)	10^6^ (Δ*g*/*f*)	10^6^ *ϵ c*_20_	10^6^δ*a*/*a*
*T*/K = 210.7400								
11	1473.6870	1474.0150	1473.7963	0.1392	0.1233	10.8	−564.0	533.6
12	3285.6832	3286.0910	3285.8191	0.1594	0.1428	5.0	−560.8	530.6
13	5004.5941	5005.1579	5004.7820	0.1909	0.1708	4.0	556.0	526.0

*T*/K = 224.5520								
11	1473.3901	1473.7176	1473.4993	0.1404	0.1244	10.8	−563.2	532.9
12	3285.0222	3285.4294	3285.1579	0.1608	0.1441	5.1	−560.1	530.0
13	5003.5882	5004.1509	5003.7758	0.1922	0.1724	4.0	−555.0	525.1

*T*/K = 238.6638								
11	1473.0799	1473.4069	1473.1889	0.1415	0.1256	10.8	−562.5	532.2
12	3284.3317	3284.7381	3284.4672	0.1618	0.1455	5.0	−559.1	529.0
13	5002.5368	5003.0984	5002.7240	0.1936	0.1739	3.9	−554.0	524.2

*T*/K = 252.9801								
11	1472.7597	1473.0862	1472.8685	0.1425	0.1267	10.7	−561.7	531.5
12	3283.6186	3284.0246	3283.7539	0.1632	0.1468	5.0	−558.7	528.6
13	5001.4510	5002.0119	5001.6380	0.1952	0.1755	3.9	−553.5	523.7

*T*/K = 273.1600								
11	1472.2981	1472.6239	1472.4067	0.1440	0.1283	10.7	−560.7	530.5
12	3282.5908	3282.9959	3282.7258	0.1648	0.1486	4.9	−557.6	527.6
13	4999.8863	5000.4459	5000.0728	0.1971	0.1777	3.9	−552.3	522.6

*T*/K = 287.4650								
11	1471.9652	1472.2905	1472.0736	0.1450	0.1294	10.6	−560.0	529.8
12	3281.8494	3282.2537	3281.9842	0.1659	0.1499	4.9	−556.7	526.7
13	4998.7574	4999.3161	4998.9436	0.1984	0.1792	3.8	−551.6	521.9

*T*/K = 302.9301								
11	1471.6001	1471.9249	1471.7084	0.1461	0.1306	10.5	−559.2	529.1
12	3281.0362	3281.4399	3281.1708	0.1672	0.1513	4.9	−556.0	526.1
13	4997.5193	4998.0772	4997.7053	0.1998	0.1809	3.8	−550.9	521.3

**Table A2:** Mean values and relative standard deviations of (*u*/*a*_0_) for argon from 5 modes

*p*/kPa		〈*u*/*a*_0_〉/s^−1^	10^6^ *σ*
	*T* = 302.9166 K	*a*_0_ = 88.94344 mm	

556.997		3649.8707	0.9
556.997		3649.8707	0.9
499.559		3649.3006	0.8
450.125		3648.8134	0.8
400.000		3648.3241	0.7
349.777		3647.8375	0.6
299.646		3647.3560	0.6
299.646		3647.3562	0.6
249.957		3646.8834	0.6
199.729		3646.4091	0.7
149.930		3645.9430	0.6
149.930		3645.9440	0.5
104.875		3645.5260	0.3
74.848		3645.2492	0.6
49.898		3645.0222	0.4
24.974		3644.7939	0.5

	*T* = 293.1300 K	*a*_0_ = 88.92949 mm	

537.608		3590.1231	0.6
483.268		3589.6591	0.6
430.165		3589.2111	0.4
375.878		3588.7586	0.5
322.145		3588.3150	0.5
267.906		3587.8722	0.6
267.906		3587.8725	0.7
213.940		3587.4378	0.7
159.913		3587.0067	0.5
105.775		3586.5809	0.3
105.775		3586.5811	0.3
75.145		3586.3436	0.5
49.763		3586.1472	0.5
24.944		3585.9553	0.4

	*T* = 273.1600 K	*a*_0_ = 88.90141 mm	

500.233		3464.8475	1.1
451.426		3464.5995	0.9
399.112		3464.3411	0.7
349.798		3464.1033	0.7
300.659		3463.8680	0.6
250.247		3463.6343	0.7
200.970		3463.4087	0.4
150.375		3463.1832	0.5
127.709		3463.0844	0.5
100.288		3462.9658	0.4
100.288		3462.9657	0.5
74.917		3462.8563	0.3
50.006		3462.7512	0.6
25.030		3462.6469	0.4

	*T* = 253.1500 K	*a*_0_ = 88.87387 mm	

470.069		3334.3519	0.4
422.800		3334.3271	0.4
375.753		3334.3074	0.3
328.542		3334.2965	0.3
282.174		3334.2842	0.3
234.896		3334.2783	0.4
234.761		3334.2796	0.4
188.108		3334.2787	0.3
140.855		3334.2838	0.3
105.364		3334.2902	0.4
71.751		3334.2987	0.3
49.850		3334.3070	0.4
24.921		3334.3162	0.4

	*T* = 234.3156 K	*a*_0_ = 88.84858 mm	

480.511		3206.1452	0.4
480.511		3206.1468	0.4
450.963		3206.2935	0.6
400.725		3206.5511	0.2
350.927		3206.8116	0.7
299.286		3207.0896	0.4
250.141		3207.3522	0.6
200.101		3207.6324	0.4
150.081		3207.9147	0.2
100.575		3208.2014	0.6
74.711		3208.3512	0.6
49.933		3208.4986	0.5
25.010		3208.6469	0.6

	*T* = 217.0950 K	*a*_0_ = 88.82605 mm	

357.289		3085.0067	0.3
326.549		3085.3776	0.5
291.181		3085.8025	0.5
255.374		3086.2390	0.5
220.638		3086.6611	0.5
185.789		3087.0880	0.6
149.955		3087.5313	0.6
149.955		3087.5317	0.8
115.584		3087.9581	0.5
102.231		3088.1273	0.6
102.144		3088.1284	0.6
74.809		3088.4694	0.3
50.272		3088.7773	0.3
24.969		3089.0984	0.3

**Table A3:** Mean values and standard deviations of (*u*/*a*_0_) for xenon corrected for the virtual leak

*p*/kPa		〈*u*/*a*_0_〉/s^−1^	10^6^*σ*
	*T* = 302.9166 K	*a*_0_ = 88.94344 mm	

302.569		1998.9335	0.5
269.851		2000.1990	0.6
239.836		2001.3569	0.7
199.948		2002.8931	0.7
150.100		2004.8092	0.9
133.515		2005.4456	1.0
105.227		2006.5302	0.8
79.949		2007.4980	0.8
54.958		2008.4542	0.7
44.964		2008.8364	0.6
33.065		2009.2912	0.4

	*T* = 273.1600 K	*a*_0_ = 88.90141 mm	

299.353		1893.8741	0.6
299.118		1893.8870	0.6
270.053		1895.4906	0.7
239.722		1897.1588	0.7
239.531		1897.1690	0.7
210.107		1898.7816	0.7
179.899		1900.4307	0.8
150.130		1902.0510	0.8
119.991		1903.6854	0.8
89.940		1905.3097	0.7
60.019		1906.9218	0.6
45.059		1907.7253	0.5
29.906		1908.5384	0.4
